# AP4 positively regulates LAPTM4B to promote hepatocellular carcinoma growth and metastasis, while reducing chemotherapy sensitivity

**DOI:** 10.1002/1878-0261.12171

**Published:** 2018-02-06

**Authors:** Yue Meng, Lu Wang, Jianjun Xu, Qingyun Zhang

**Affiliations:** ^1^ Department of Clinical Laboratory Key Laboratory of Carcinogenesis and Translational Research (Ministry of Education) Peking University Cancer Hospital & Institute Beijing China

**Keywords:** AP4, C‐MYC, hepatocellular carcinoma, LAPTM4B, transcription

## Abstract

Polymorphisms of the lysosomal‐associated protein transmembrane‐4 beta (LAPTM4B) gene are related to various forms of tumour susceptibility, which led us to hypothesize that some unique transcription factors targeting this polymorphism region may affect the biological function of LAPTM4B in tumour progression. In this study, we found that the transcription factor AP4 directly binds to the polymorphism region of the LAPTM4B gene promoter and induces its transcription. In addition, we demonstrated that AP4 promotes hepatocellular carcinoma (HCC) cell proliferation and metastasis and depresses chemotherapy sensitivity via LAPTM4B by activating the PI3K/AKT signalling pathway and caspase‐dependent pathway. Interestingly, we found that AP4 could not only regulate LAPTM4B by directly binding to the promoter, but also be regulated via a positive feedback mechanism involving LAPTM4B acting on c‐myc. Finally, we showed that AP4 and LAPTM4B are highly coexpressed in HCC tissues, and their coexpression may be a marker of poor prognosis. These findings provide evidence of the expression and functional coupling between AP4 and LAPTM4B and shed light on the regulation of LAPTM4B and its function in liver cancer.

**Abbreviations**
AKT: protein kinase B
AP4: activator protein 4
CREB‐1: cAMP‐responsive element‐binding protein‐1
EGFR: epidermal growth factor receptor
EMSA: electrophoretic mobility shift assays
EMT: epithelial–mesenchymal transition
HCC: hepatocellular carcinoma
LAPTM4B: lysosomal‐associated protein transmembrane‐4 beta
p‐GSK3β: phosphorylation‐glycogen synthase kinase‐3β
PI3K: phosphatidylinositol 3 kinase
qPCR: quantitative reverse transcriptase polymerase chain reaction
SHP1: Src homology region 2 domain‐containing phosphatase‐1
SOE: splice overlap extension

## 1 Introduction

Lysosomal‐associated protein transmembrane‐4 beta (LAPTM4B) is a high‐expression gene in hepatocellular carcinoma (HCC) cells identified by fluorescent‐differential display (Shao *et al*., 2003; Liu *et al*., 2000; Meng *et al*., 2016). Previous studies have identified two alleles of the LAPTM4B gene, which are differentiated from one another by an extra tandem arranged 19‐bp sequence at the 5′ untranslated region (5′UTR) in the first exon in allele*2. LAPTM4B allele*2 is strongly associated with the susceptibility to HCC (Wang *et al*., 2012; Zhai *et al*., 2012), breast cancer (Fan *et al*., 2012), colorectal carcinoma (Cheng *et al*., 2008), gallbladder carcinoma (Yang *et al*., 2012), ovarian cancer (Xu *et al*., 2012), non‐small cell lung cancer (Tang *et al*., 2014), gastric cancer (Liu *et al*., 2007) and others (Meng *et al*., 2011; Zhang *et al*., 2014). However, the mechanisms that underlie the tumour susceptibility of patients with LAPTM4B allele*2 are still unclear.

As the only difference between the two LAPTM4B alleles is the number of 19‐bp sequences in the 5′UTR, we hypothesized that some unique transcription factors that act in this polymorphism region may affect the biological function of LAPTM4B in tumour progression. In a previous study, we identified transcription factors that act upstream and downstream of the 19‐bp sequence and showed that sp1 (Xue *et al*., 2007) acts upstream and cAMP‐responsive element‐binding protein‐1 (creb‐1; Zhang *et al*., 2013) acts downstream to increase LAPTM4B expression. In this study, we determined that the protein activating protein‐4 (AP4), a member of the basic helix‐loop‐helix leucine‐zipper (bHLH‐LZ) transcription factor family, which exclusively forms homodimers that bind to the E‐box motif CAGCTG (Hu *et al*., 1990), directly binds to the 19‐bp sequence of the LAPTM4B gene promoter and induces its transcription. AP4 promotes HCC cell proliferation, migration and invasion and reduces chemotherapy sensitivity via LAPTM4B by activating the phosphoinositide 3‐kinase (PI3K)/protein kinase B (AKT) signalling pathway and caspase‐dependent pathway. In addition, we examined the relationship between c‐myc, AP4 and LAPTM4B and found an atypical AP4‐c‐myc‐LAPTM4B‐positive feedback loop. Finally, we showed that AP4 and LAPTM4B were highly coexpressed in HCC tissues, and their coexpression might be a marker of poor prognosis. These findings indicate an expression and functional coupling between AP4 and LAPTM4B and shed light on the regulation of LAPTM4B and its function in liver cancer.

## 2 Materials and methods

### 2.1 Cells and culture conditions

Human HCC cells, including BEL‐7402, Huh7, HepG2 and LO2 cells, were maintained in Roswell Park Memorial Institute‐1640 medium (HyClone, Logan, UT, USA) supplemented with 10% fetal bovine serum and antibiotics. All cells were cultured at 37 °C, 5% CO_2_. Huh7 and HepG2 cells were purchased from CellCooK (Guangzhou, China). Bel‐7402 cells were purchased from COBIOER (Nanjing, China). Our laboratory maintained the LO2 cell line.

### 2.2 Screening transcription factors of promoter region

The 5′‐genomic sequence of the human LAPTM4B gene was obtained from GenBank, and the promoter was predicted by ENSEMBLE (http://www.ensembl.org/index.html). The transcription start site of the sequence was termed +1. A 501‐bp (−200 to +301) and a 520‐bp (−200 to +320) double‐stranded DNA sequence, which were predicted to be the LAPTM4B allele*1 and LAPTM4B allele*2 promoter region, were chemically synthesized and digested from the T vector by XhoI and HindIII digestion. Transcription factor screening was performed using the TF activation profiling plate array II (FA‐1002; Signosis, Santa Clara, CA, USA) and the online transcription factor binding prediction database.

### 2.3 Luciferase reporter assay

A 283‐bp fragment with one AP4 binding site (CAGCTG) was mutated by splice overlap extension (SOE)‐PCR using a modified reverse primer spanning the AP4 binding site (Table S3) and was cloned into a Hind III‐Xho I‐restricted pGL3‐Basic vector to generate pGL3‐mut+10‐+292 (named Mut+10‐+292, mutation sequence: TCAACT). The plasmids containing the first mutated AP4 binding site (named Mut1+10‐+311, mutation sequence: TCAACT), the second mutated AP4 binding site (named Mut2+10‐+311, mutation sequence: TCAACT) and both mutated AP4 binding sites (named Mut double+10‐+311, mutation sequence: TCAACT) were made by SBS Genetech CO., Ltd. (Beijing, China). Our laboratory created the other seven truncated plasmids (Zhang *et al*., 2013). All luciferase reporter plasmids were identified by enzyme digestion and sequenced (Figs S7 and S8). For reporter assays, BEL‐7402 cells, HepG2 cells and Huh7 cells were transfected with these plasmids (Fig. S9). Luciferase activity was measured 48 h after transfection using the dual‐luciferase reporter assay system (E1910; Promega, Madison, WI, USA).

### 2.4 Electrophoretic mobility shift assays (EMSAs) and Supershift assays

Double‐stranded DNA oligonucleotides containing the AP4 binding site found in the LAPTM4B promoter (sense: 5′‐GGAGCTCCAGCAGCTGGCTGGAGCC‐3′), as well as mutated AP4 oligonucleotides (sense: 5′‐GGAGCTCCAGTCAACTGCTGGAGCC‐3′), were labelled with biotin (Viagene Biotech Inc, Changzhou, China). We performed the assay as previously described (Zhang *et al*., 2013).

For the supershift assay, 1 μg anti‐AP4 antibodies (Santa Cruz, Dallas, TX, USA, sc‐18593X) and normal goat IgG were pre‐incubated with 4.5 μg nuclear extracts with binding buffer for 20 min at room temperature before the addition of oligonucleotide probes.

### 2.5 ChIP‐qPCR assays

DNA·protein complexes were immunoprecipitated from cells using a ChIP assay kit (Millipore, Bedford, MA, USA) according to the manufacturer's protocol with anti‐AP4 antibodies (Santa Cruz; sc‐18593X) or normal IgG, with the latter serving as a control for nonspecific DNA binding. Immunoprecipitated DNA was purified and then analysed by real‐time PCR. The real‐time PCR primers for AP4 were as follows: (F) 5′‐GCCAGATGAAAGAAGGAAGG‐3′ and (R) 5′‐ATGTGACCCGAGTCCGTGA‐3′. The real‐time PCR primers for negative control region were 5′‐ CTCGGTTGGTTGGGCAGG‐3′ and (R) 5′‐GAGTCTCGCTCTTGTCGC‐3′. The results of the real‐time PCR were analysed by $2^{-\Delta \Delta C_{T}}$.

### 2.6 DNA extraction from cell samples and PCR

Genomic DNA was extracted from cells and paraffin‐embedded sections using a DNA extraction kit (Axygen, Union City, CA, USA). For each sample, the genotype of LAPTM4B was identified by PCR using the following primers: 5′‐GCCGACTAGGGGACTGGCGGA‐3′ (P1 forward) and 5′‐CGAGAGCTCCGAGCTTCTGCC‐3′ (P2 reverse). The PCR mixture (25 μL) contained 0.5 U *Taq* polymerase (TIANGEN, Beijing, China) and 1 μL template DNA at a final concentration of 100 ng·μL^−1^. Human β‐actin was used as an internal positive control using the following primers: 5‐TCACCAACTGGGACGACAT‐3 (forward), 5‐AGGTAGTCAGTCAGGTCCCG‐3 (reverse). The PCR products were analysed by electrophoresis in a 2.5% agarose gel and visualized with ethidium bromide.

### 2.7 Transient transfection with siRNA and treatment with the c‐myc inhibitor 10058‐F4

The siRNA sequences targeting AP4 (Table S4) used in this study were synthesized by QIAGEN (1027416; Germantown, MD, USA). c‐myc siRNA #1, #2 and #3 were synthesized by RiboBio (stQ0003660‐1; Guangzhou, China). The c‐myc inhibitor 10058‐F4 was purchased from Selleck (Houston, TX, USA). Twenty‐four hours prior to transfection, HepG2, BEL‐7402, LO2 and Huh7 cells were plated onto a 6‐well plate (Nest, Biotech, WuXi, China) at 50–70% confluence. siRNA was then transfected at a working concentration of 50 nm using Lipofectamine^®^ 3000 Transfection Reagent (L3000015, Thermo Fisher Scientific Inc, Waltham, MA, USA).

### 2.8 Reverse transcriptase PCR and quantitative reverse transcriptase PCR

RNA samples were extracted using the TRIzol reagent (15596‐026; Invitrogen, Waltham, MA, USA). Reverse transcription was then performed using the FastQuant RT kit (KR106; TIANGEN Biotech, Beijing, China). For quantitative reverse transcriptase PCR, we used the FastFire qPCR PreMix SYBR Green kit (FP207; TIANGEN Biotech). The primer sequences are shown in Table S5.

### 2.9 Establishment of HCC cell lines with stable LAPTM4B overexpression and stable TFAP4 knockdown

Lentiviral (Lenti‐OE™ Custom, Ubi‐MCS‐3FLAG‐SV40‐EGFP‐IRES‐puromycin) particles carrying the full‐length LAPTM4B gene coding sequence and lentiviral (Lenti‐KO™ Custom, hU6‐MCS‐Ubiquitin‐EGFP‐IRES‐puromycin) particles carrying AP4 shRNA (sense, 5′‐GGUGCCCUCUUUGCAACAU‐3′) were purchased from GeneChem (Shanghai, China). BEL‐7402 and Huh7 HCC cells were infected with recombinant lentiviral particles according to the manufacturer's protocol.

### 2.10 Antibodies and western blot

Antibodies used for western blot analysis are shown in Table S6.

### 2.11 Cell proliferation analysis

Cell Counting Kit‐8 (CCK‐8; Dojindo, Kumamoto, Japan) was used to evaluate cell proliferation. For cell proliferation, 1 × 10^3^ cells were seeded into a 96‐well plate in triplicate for each condition. All cells were incubated for 4 days. CCK‐8 solution (10 μL) was added to each well at the indicated time point, and cells were incubated for 2 h at 37 °C. Cell proliferation was assessed by measurement of the optical density at 450 nm. Experiments were performed three times.

### 2.12 *In vitro* cell migration and invasion assays

*In vitro* cell migration and invasion assays were performed according to a previous description (Cheng *et al*., 2015). Experiments were performed three times.

### 2.13 Colony formation assay

Cells were plated in small culture plates (*d* = 6 cm) at 500 cells/well with three wells per cell group. After incubation for 12 days at 37 °C, cells were fixed in 4% paraformaldehyde for 5 min at room temperature and stained with crystal violet for 5 min. The number of colonies containing more than 50 cells was counted under a microscope. The colony formation efficiency was calculated as (number of colonies/number of cells inoculated) × 100%.

### 2.14 Tumorigenesis in NOD/SCID mice

We established xenografts in NOD/SCID mice with the stable Huh7 cells with LAPTM4B overexpression, Huh7 cells with LAPTM4B overexpression and TFAP4 knockdown, Huh7 cells with TFAP4 knockdown and control cells. Here, 3 × 10^6^ cells were subcutaneously implanted into the dorsal flank of female NOD/SCID mice (*n* = 5 per group, 5 weeks of age). The tumour volumes were measured once every 1 week and calculated as 1/2 × (length) × (width). All mice were sacrificed at 7 weeks after the inoculation, and the tumour volumes and tumour weights were recorded. The experiment was approved by the Institutional Animal Welfare Committee.

### 2.15 Cell cycle assay

Cells were collected, fixed in 70% cold ethanol for at least 4 h at −20 °C and stained with 50 μg·mL^−1^ propidium iodide (BD Biosciences, New York, NY, USA) at room temperature for 15 min in the dark, and cell cycle analysis was performed using the FACS Calibur system (BD Biosciences) and analysed by modfit 3.0 software (Verity Software House, Topsham, ME, USA).

### 2.16 Cytotoxicity assays

Cells were plated in triplicate at a density of 10 000 cells/well in 96‐well plates and treated with the indicated concentrations of doxorubicin and paclitaxel. At the indicated time points, Cell Counting Kit‐8 (CCK‐8; Dojindo) was used to evaluate cell viability. Experiments were performed three times.

### 2.17 Annexin V apoptosis assay

Cell apoptosis analysis was conducted by APC and 7‐AAD (BD Biosciences) staining for 15 min at room temperature in the dark, followed by flow cytometry analysis within 1 h (BD Biosciences). Cell apoptosis was analysed by cflowplus software (BD Biosciences).

### 2.18 Immunohistochemistry

Immunohistochemistry analysis of AP4 (ab28512; Abcam, Cambridge, MA, USA), LAPTM4B (bs‐6542R Bioss, Boston, MA, USA) and Ki‐67 (ZA‐0502; ZSGB‐BIO, Beijing, China) was performed in HCC tissues according to a previous description (Li *et al*., 2017). Stained tissue sections were evaluated separately by two pathologists without any knowledge of the clinical data. For cytoplasmic staining, scoring was assessed based on the sum of cytoplasm staining intensity and the percentage of positive staining areas of cells. The staining intensity was scored as 0 (negative), 1 (weak), 2 (medium) and 3 (strong) and the percentage of positive staining areas of cells was defined as a scale of 0–3, where 0 represents < 10%, 1 was 10–25%, 2 was 26–75% and 3 was ≥ 76%. For nuclear staining, the score was defined according to the sum of nuclear staining intensity and numbers of cells. Nuclear staining intensity score was consistent with cytoplasm and positive nuclear staining scores were defined as follows: 0 represents < 10%, 1 was 10–50%, 2 was 51–80% and 3 was ≥ 80%. For statistical analysis, scores of 0–3 or 4–6 was, respectively, considered to be low or high expression.

### 2.19 Collection of HCC specimens and ethics statement

Samples analysed included 117 paraffin‐embedded HCC specimens obtained at the time of diagnosis before any therapy from Beijing Cancer Hospital (Beijing, China). Clinical processes were approved from the Ethics Committees of Beijing Cancer Hospital. Patients provided informed consent. The pathologic stage of all specimens was confirmed according to the 2000 HCC staging system of the WHO. Follow‐up was performed until May 2017 for 117 patients. The demographic and clinicopathological features of all patients were provided in Table S7 and S8.

### 2.20 Statistical analysis

spss 13.0 (IBM, Armonk, NY, USA) was used for statistical analysis. The Pearson test was applied to examine the relationship between LAPTM4B and AP4 mRNA expression. The Spearman test was utilized to evaluate the relationship between LAPTM4B and AP4 protein expression. One‐way ANOVA was used to measure the association between tumour grades with AP4 and LAPTM4B mRNA expression level. Survival analysis was performed using the Kaplan–Meier method. Two‐tailed Student's t‐test was used for comparisons of two independent groups.

## 3 Results

### 3.1 Screening for transcription factors with binding sites located in the LAPTM4B promoter polymorphism region

**Figure 1:**
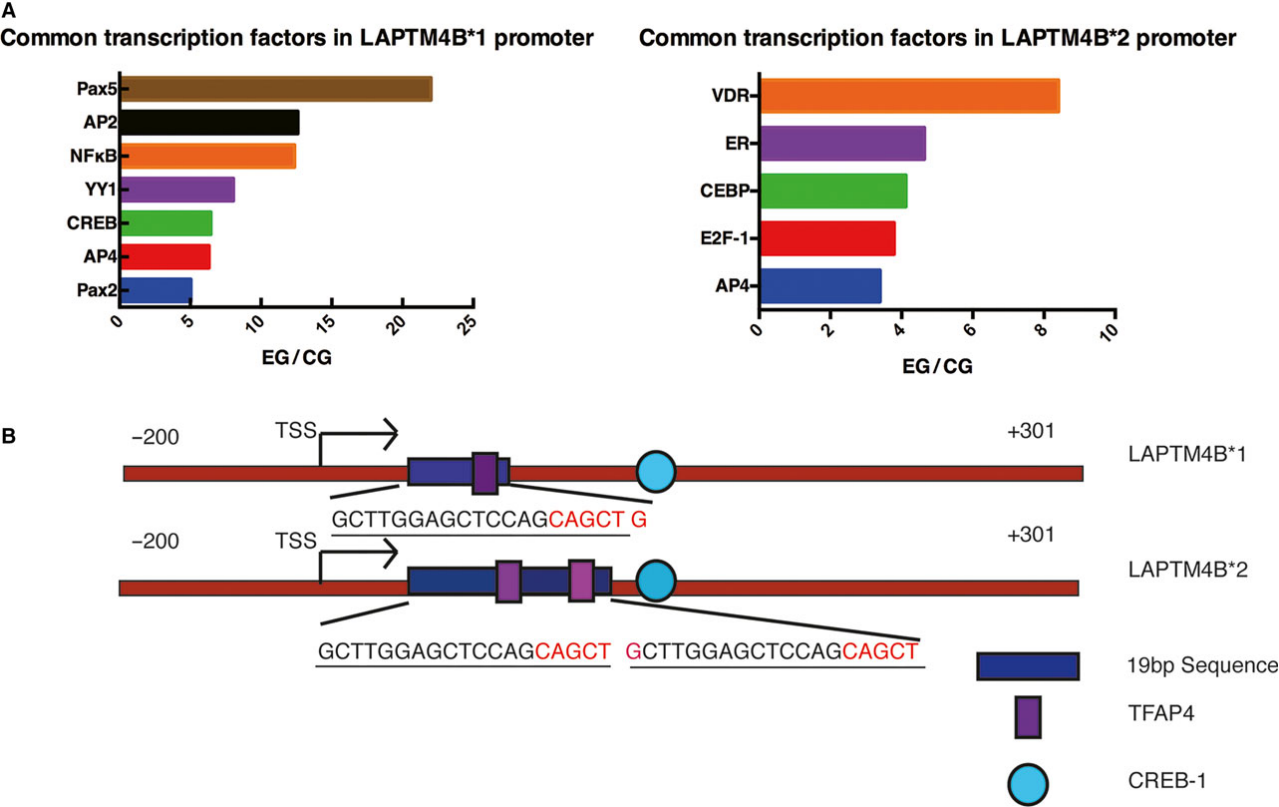
Common transcription factors defined by two assays. (A) A comparison of the results of the transcription factor activation profiling plate with those of the online prediction database revealed common transcription factors. (B) CREB‐1 is predicted to bind after the 19‐bp polymorphism region. AP4 is predicted to bind within the 19‐bp polymorphism region. The LAPTM4B*1 promoter has only one TFAP4 binding site, whereas the LAPTM4B*2 promoter has two TFAP4 binding sites in the 19‐bp polymorphism region.

To screen for transcription factors that act on the LAPTM4B promoter region, two methods were used to investigate the LAPTM4B allele*1 promoter, a 500‐bp (−200 to +301) sequence, and the LAPTM4B allele*2 promoter, a 519‐bp (−200 to +320) sequence. A comparison of the results of the online prediction database (Tables S1 and S2) with those of the transcription factor activation profiling plate (Fig. S1A,B) revealed many common transcription factors (Fig. 1A). We specifically focused on two transcription factors with binding sites located near the 19‐bp polymorphism region (Fig. 1B). CREB‐1 was predicted to bind at +159 to +166, which is located after the 19‐bp polymorphism region and has been shown to positively regulate the LAPTM4B gene (Zhang *et al*., 2013). AP4 was the only one factor predicted to bind within the 19‐bp polymorphism region in both the LAPTM4B*1 promoter and LAPTM4B*2 promoter. Moreover, the online prediction database suggested that there are two AP‐4 binding sites in the LAPTM4B allele*2, which suggests that the LAPTM4B allele*2 may bind more AP4 to affect LAPTM4B transcription activity (Table S2). Considering the above observations, AP4 may play a key role in the transcription regulation of the LAPTM4B gene.

### 3.2 AP4 positively regulates LAPTM4B gene transcription by binding to the LAPTM4B gene promoter and more strongly affects LAPTM4B allele*2 transcription activity

**Figure 2:**
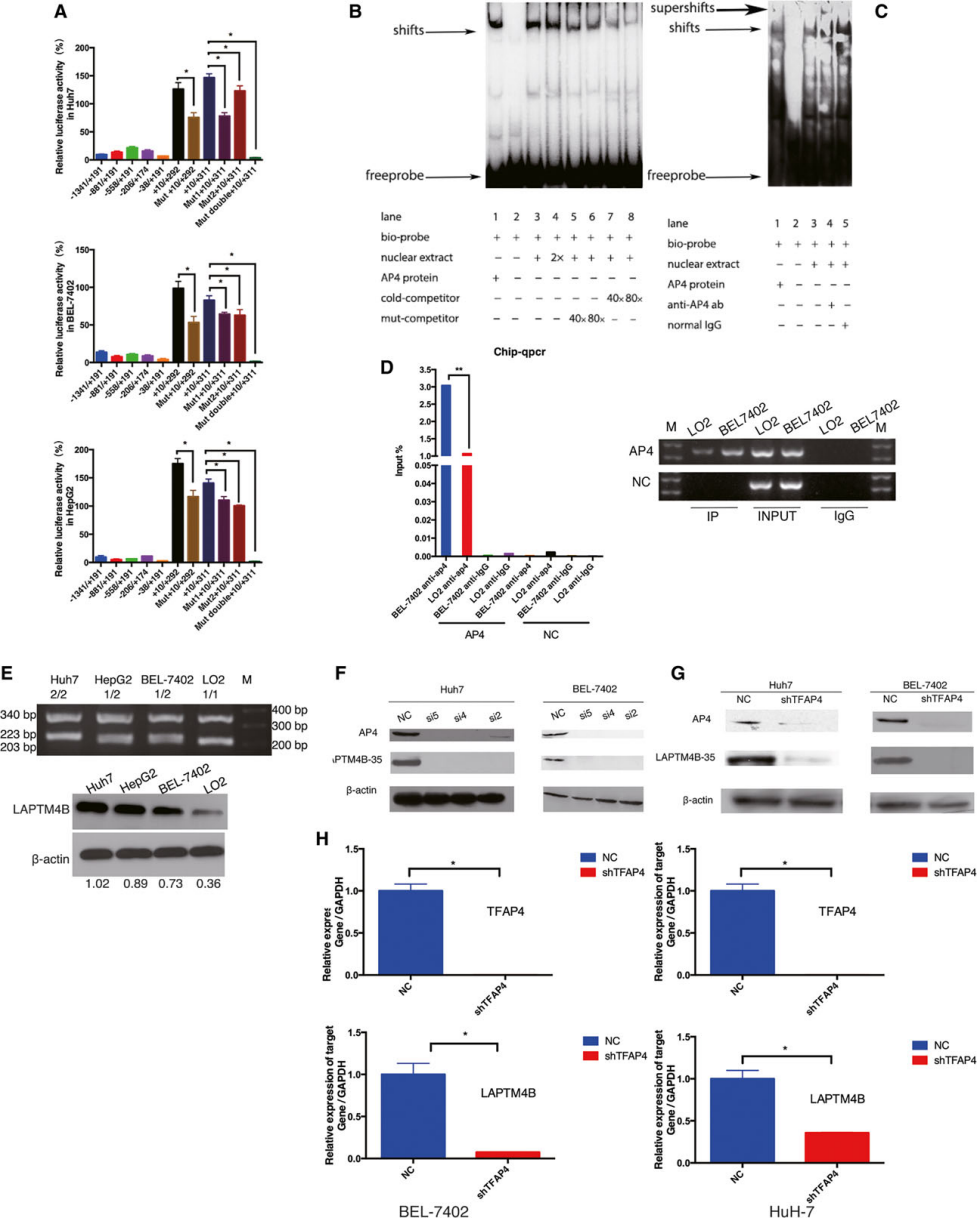
AP4 positively regulates LAPTM4B gene transcription by binding to the LAPTM4B gene promoter. (A) Transcription activity associated with LAPTM4B promoter regions. Dual‐luciferase assays were performed in three HCC cell lines. Data are presented as the mean ± SD. All experiments were repeated three times (*P < 0.05). (B) The ability of the transcription factor AP4 to bind the LAPTM4B promoter. DNA·protein complexes were separated by electrophoresis and detected as ‘shifts’ from the position of the free probe. (C) The additional goat anti‐AP4 antibody produced a visible ‘supershift’ and partially disrupted the ‘shift’. (D) ChIP assay to determine the binding of AP4 to the LAPTM4B promoter in BEL‐7402 and LO2 cells. AP4 means target fragment and NC means negative control region on LAPTM4B promoter. (E) LAPTM4B expression in different cell lines with different genotypes of LAPTM4B. M is DNA marker. LO2 genotype is LAPTM4B*1/1. BEL‐7402 and HepG2 genotype are LAPTM4B*1/2. Huh7 genotype is LAPTM4B* 2/2. 204 bp is the LAPTM4B*1 allele sequence, 223 bp is the LAPTM4B*2 allele sequence, 340 bp is the β‐actin sequence. (F) Huh7 and BEL‐7402 cells were transfected with three siRNA targeting AP4 to verify that knockdown of AP4 could suppress LAPTM4B‐35 expression. (G, H) The stable AP4 knockdown HCC cell lines showed that the expression of LAPTM4B was suppressed at both the protein and mRNA levels (*P < 0.05). All experiments were repeated three times.

To explore whether AP4 could play a part in transcription regulation, we first transfected three HCC cell lines with eleven plasmids containing different LAPTM4B promoter regions and examined the relative luciferase activity (Fig. 2A). The fragment +10 to +292, which includes one AP4 binding site and corresponds to the LAPTM4B allele*1, and the fragment +10 to +311, which included two AP4 binding sites and corresponds to LAPTM4B allele*2, showed stronger luciferase activity than any of the other constructs. The transcriptional activity of the constructs containing mutated AP4 binding sites was significantly lower than that of the constructs containing the wild‐type binding sites, regardless of whether the mutated binding site was in the first or second AP4 binding site. The construct containing mutations of both binding sites had the lowest transcriptional activity. These results suggest that AP4 may have a greater effect on the transcription activity of the LAPTM4B allele*2; however, there was no difference in the effect of AP4 found between the first binding site and the second binding site in the LAPTM4B allele*2.

To determine whether the transcription factor AP4 can bind the core region of the LAPTM4B promoter *in vivo* and *in vitro*, an EMSA and chromatin immunoprecipitation‐quantitative polymerase chain reaction (ChIP‐qPCR) were performed. In the EMSA (Fig. 2B) and supershift assay (Fig. 2C), AP4 was shown to bind to the LAPTM4B gene specifically *in vitro*. Similarly, the CHIP‐qPCR results suggested that more AP4 protein was bound to LAPTM4B in HCC cells (BEL‐7402 cells) with the LAPTM4B 1/2 gene phenotype (Fig. S2) than to that in normal hepatocyte cells (LO2 cells) with the LAPTM4B 1/1 phenotype (Fig. 2D). Moreover, it is suggested that the expression of LAPTM4B in HCC cell line with LAPTM4B 2/2 phenotype (Huh7 cells) was higher than that in HCC cell line with LAPTM4B 1/1 phenotype (LO2 cells) (Fig. 2E). Thus, these results indicate that the LAPTM4B allele*2 binds more AP4 than the LAPTM4B allele*1 in its promoter region, suggesting that AP4 may have a greater effect on the transcription activity of the LAPTM4B allele*2.

**Figure 3:**
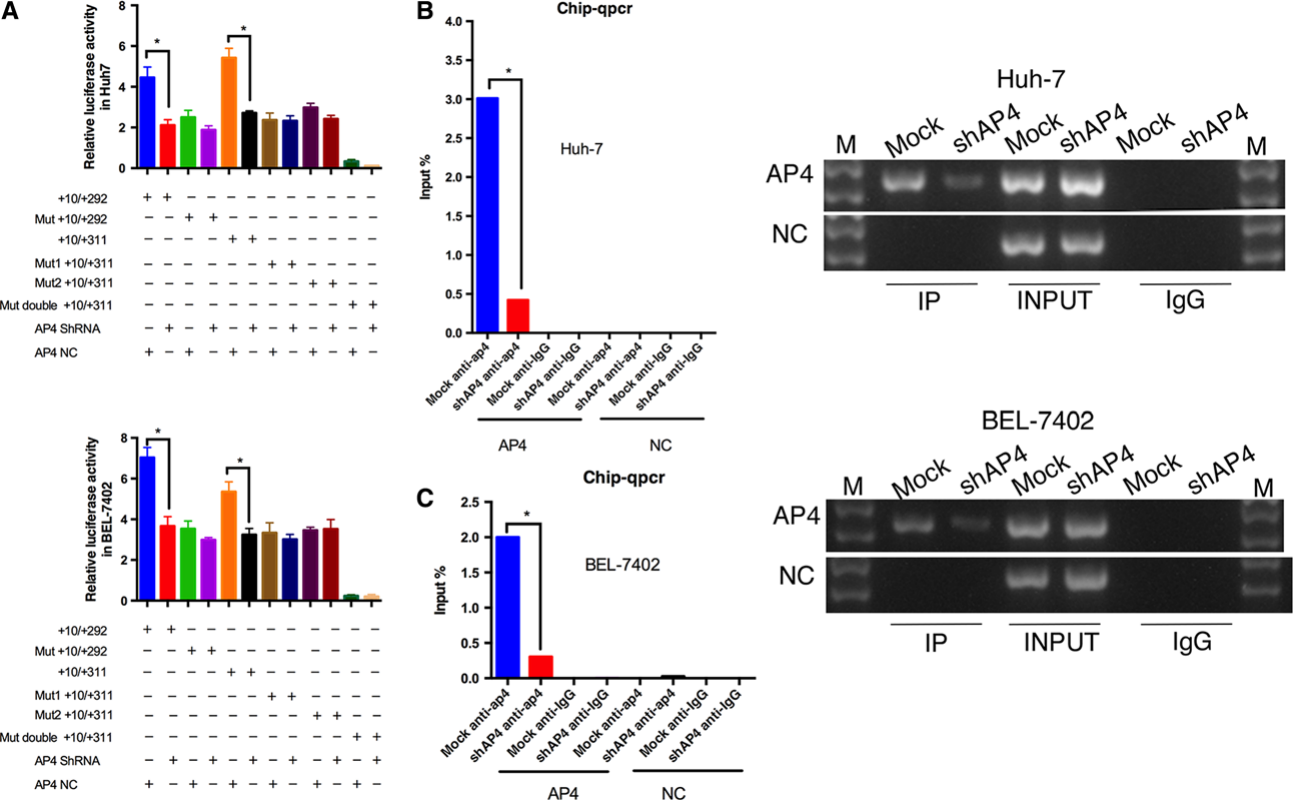
Regulatory effects of AP4 knockdown on LAPTM4B expression in ChIP and luciferase reporter assays. (A) Plasmids with wild‐LAPTM4B promoter and plasmids with mutation‐LAPTM4B promoter were transfected into AP4 stable knockdown cells and control cells (*P < 0.05). (B) ChIP assay to determine the binding of AP4 to the LAPTM4B promoter in AP4 stable knockdown cells and mock cells. AP4 means AP4 binding fragment, and NC means negative control region on LAPTM4B promoter (*P < 0.05).

To elucidate the influence of AP4 on LAPTM4B transcription, three different siRNA were used to knockdown AP4. The cells transfected with siRNA targeting TFAP4 not only expressed less AP4 than the normal control (NC) group but also expressed less LAPTM4B‐35 protein (Fig. 2F). Two HCC cell lines with AP4‐stable knockdown also demonstrated that knockdown of AP4 negatively affected LAPTM4B transcription at both the protein and mRNA levels (Fig. 2G,H). The regulatory effect of AP4 knockdown on LAPTM4B expression was also supported by changes in AP4 transactivation using luciferase reporter assays and ChIP assay. The luciferase activity of the +10/+292 plasmid and +10/+311 plasmid was lower in AP4 stable knockdown cells (AP4 shRNA) than that in AP4 control cells (AP4 NC). At the meanwhile, the luciferase activity of Mut +10/+292 plasmid and Mut +10/+311 plasmid in AP4 stable knockdown cells (AP4 shRNA) was not different than that in AP4 control cells (AP4 NC) (Fig. 3A). Moreover, ChIP assay also showed that less AP4 protein was bound to LAPTM4B in AP4 stable knockdown cells (shAP4) than that in AP4 control cells (Mock) (Fig. 3B,C). Therefore, AP4 positively regulates LAPTM4B gene transcription by binding to the LAPTM4B gene promoter.

### 3.3 AP4 promotes hepatocellular carcinoma cell growth via LAPTM4B *in vitro* and *in vivo*

**Figure 4:**
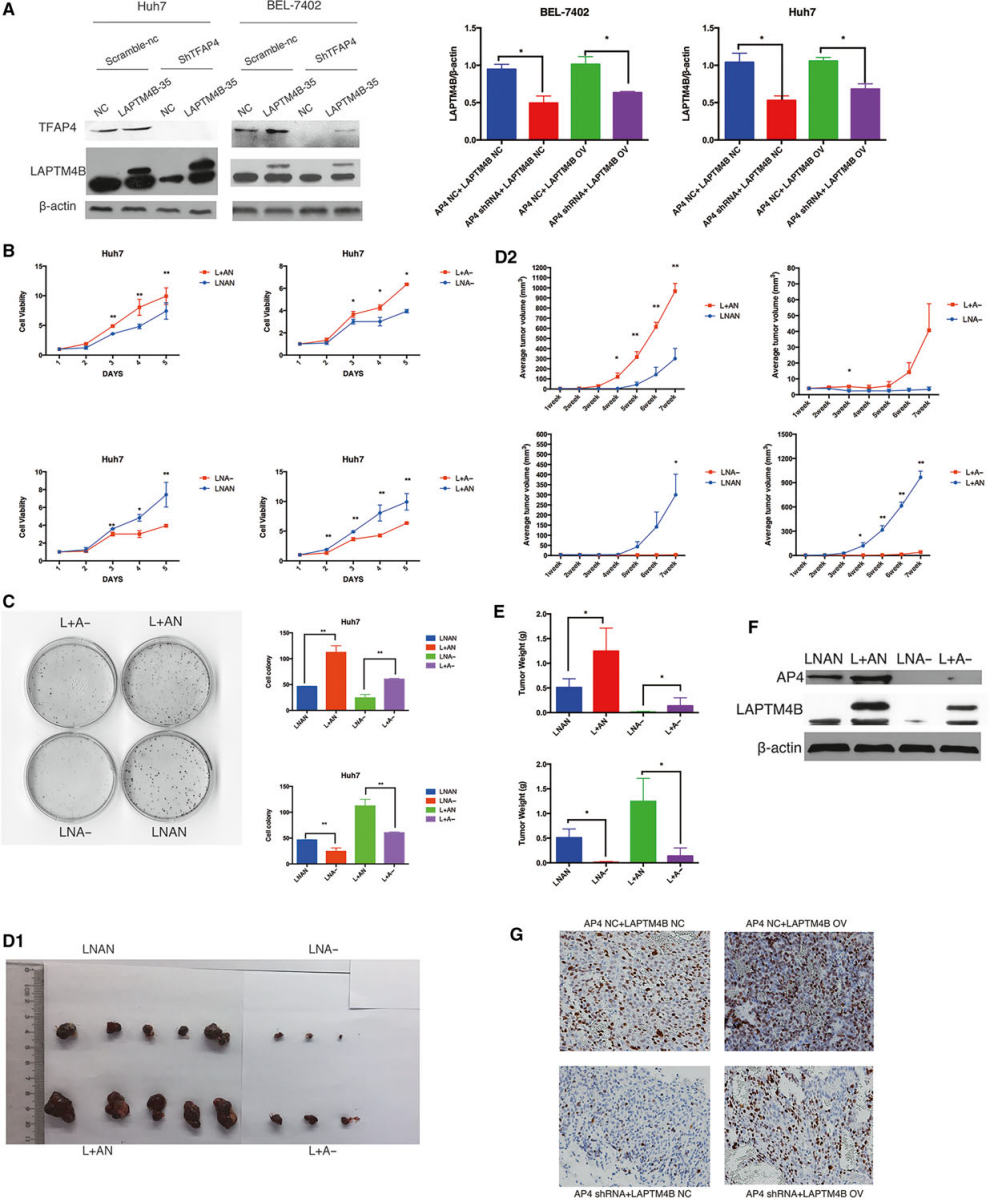
AP4 promotes HCC cell growth via LAPTM4B by affecting cell proliferation *in vitro* and *in vivo*. (A) Endogenous AP4 was silenced by shRNA, and exogenous LAPTM4B was expressed in Huh7 cells and BEL‐7402 cells. Quantification of expression was performed. (B, C) Overexpression of LAPTM4B partially but significantly rescued the cell growth arrest induced by TFAP4 knockdown in Huh7, as measured by the cell viability assay and colony formation (*P < 0.05, **P < 0.01). (D1, D2) Restoration of LAPTM4B significantly reversed the suppression of tumour growth induced by AP4 knockdown. The tumour growth curve data points are shown as the mean ± SEM (*n* = 5/group) (*P < 0.05, **P < 0.01). (E) The tumour weights were measured. (F) Intratumoral expression of LAPTM4B and AP4 were showed. (G) Restoration of LAPTM4B significantly reversed the Ki‐67 expression induced by AP4 knockdown in tumour.

Because AP4 was shown to positively regulate LAPTM4B expression, affecting transcription of LAPTM4B allele*2 to a greater extent, we speculated that AP4 may be a key regulator in the LAPTM4B transcription process, thereby affecting LAPTM4B function on tumours. AP4 and LAPTM4B have separately been shown to stimulate cell proliferation in HCC (Ge *et al*., 2014; Yang *et al*., 2010). Thus, we designed a rescue experiment to investigate the effect of AP4 on LAPTM4B and its role in cell proliferation. AP4 was knocked down by shRNA, but LAPTM4B was overexpressed in Huh7 cells and BEL‐7402 cells (Fig. 4A). A cell viability assay and colony formation assay showed that AP4 knockdown obviously inhibited cell proliferation in BEL‐7402 (Fig. S3A,B) and Huh7 cell lines (Fig. 4B,C); however, the effect was abrogated by overexpression of LAPTM4B. To assess the roles of AP4 in tumour growth *in vivo*, AP4‐mediated tumorigenesis was examined in a murine xenograft model induced by LAPTM4B and AP4 control cells (LNAN), stable Huh7 cells with LAPTM4B overexpression (L+AN), stable Huh7 cells with AP4 knockdown (LNA−), or Huh7 cells with both AP4 knockdown and LAPTM4B overexpression (L+A−) cells. Our results suggested that Huh7 cells with AP4 knockdown significantly suppressed tumour growth, but the effect was partially reversed by overexpression of LAPTM4B (Fig. 4D1,D2,E). Consistent with tumour growth results, the LNA− tumour group with lowest intratumoral (Fig. 4F) LAPTM4B expression showed the lowest Ki‐67 expression (Fig. 4G), while the L+A− group tumour partially reversed the expression of Ki‐67 by overexpression of LAPTM4B.

**Figure 5:**
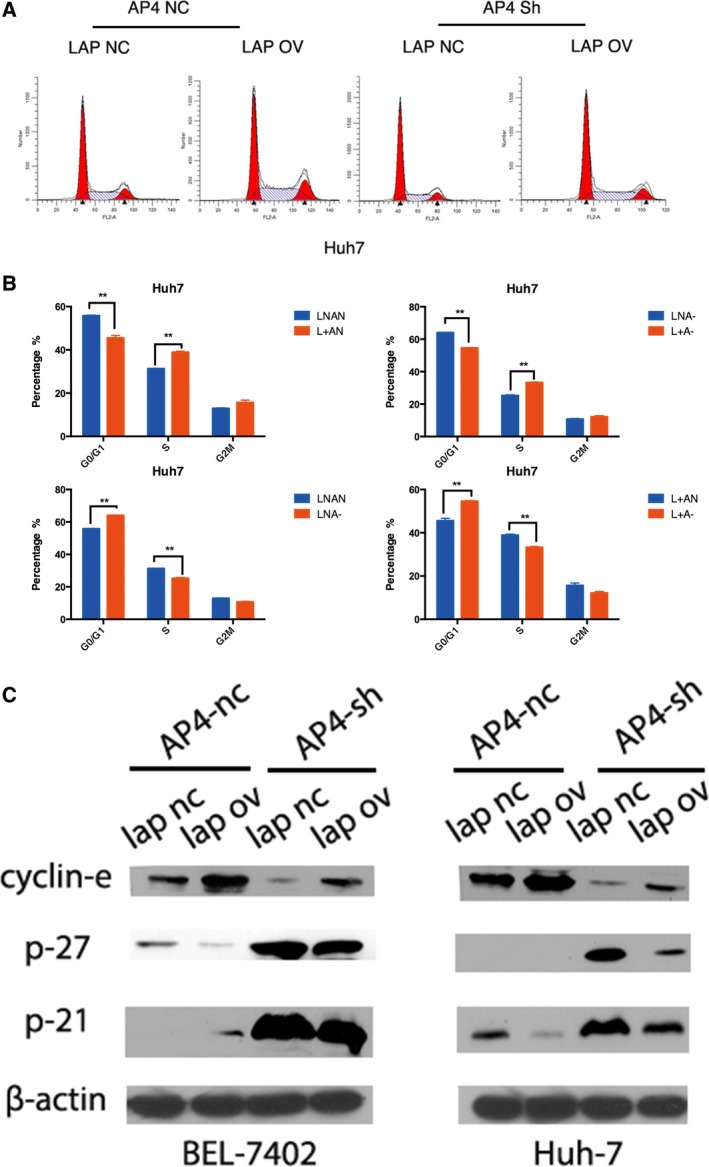
AP4 promotes HCC cell growth via LAPTM4B by affecting cell cycle *in vitro* and *in vivo*. (A, B) Restoration of LAPTM4B significantly reversed the cycle arrest at the G1 phase induced by AP4 knockdown in Huh7 cells (*n* = 3, mean ± SD) (**P < 0.01). (C) The expression of cell cycle‐related proteins p21 and p27 was upregulated, and the expression of cyclin E was downregulated in Huh7 cells with stable AP4 knockdown, while restoration of LAPTM4B significantly reversed these expression levels.

To further explore the potential mechanisms responsible for the positive effect of AP4 on tumour growth via LAPTM4B in HCC cells, a cell cycle analysis was conducted. The percentage of cells accumulated in G1 phase was significantly larger in LNA− cells and smaller in L+AN cells than in LNAN cells, while there was no difference between that in L+A− and control cells (Fig. 5A,B, Fig. S3C). Consistent with cell cycle arrest at G1, cell cycle‐related proteins p21 and p27 were upregulated, and cyclin E was downregulated (Fig. 5C).

Thus, all of these results suggest that AP4 promotes HCC cell growth via LAPTM4B by affecting the cell cycle *in vitro* and *in vivo*.

### 3.4 AP4 promotes hepatocellular carcinoma cell migration and invasion via LAPTM4B

**Figure 6:**
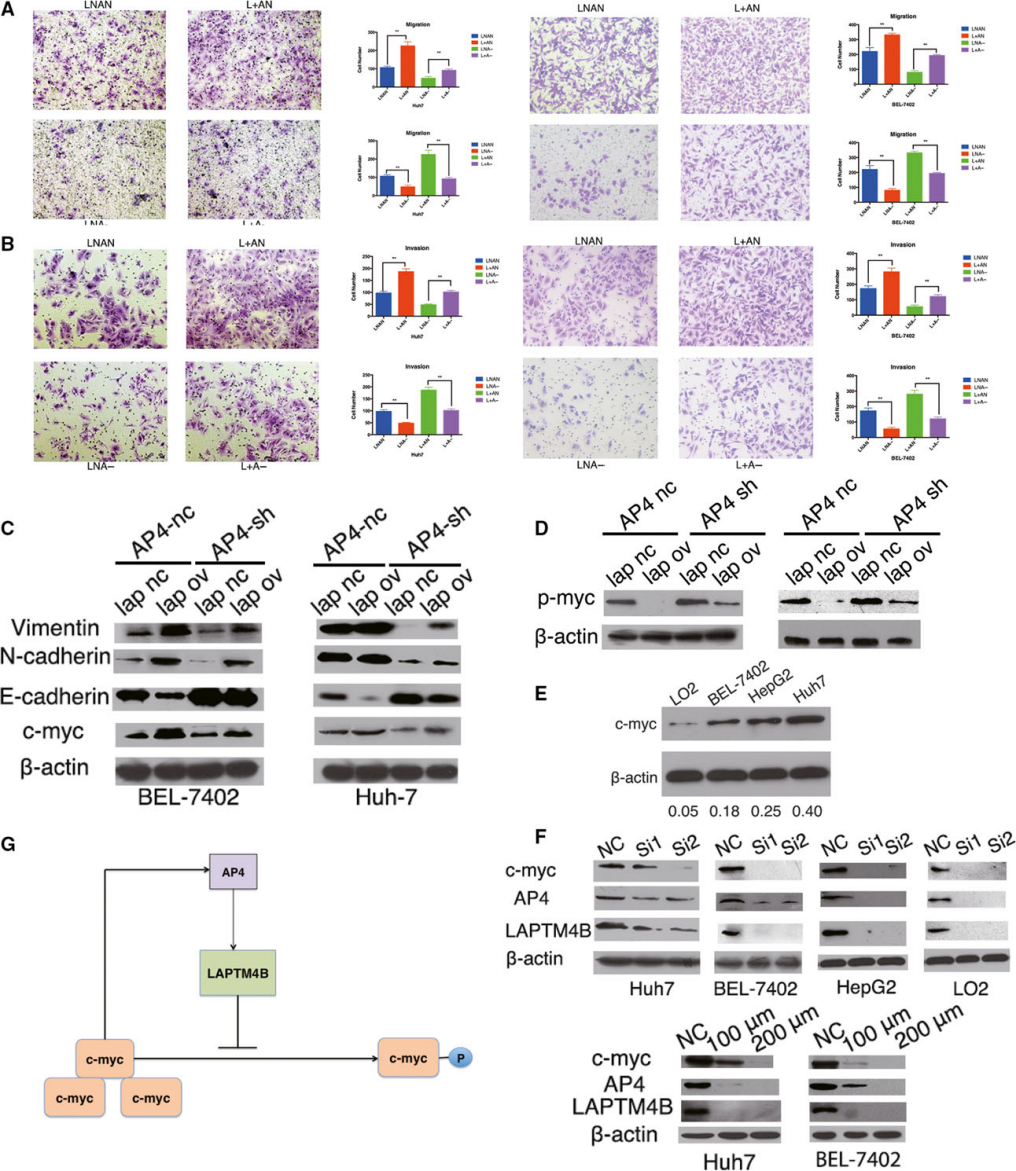
AP4 promotes HCC cell migration and invasion via LAPTM4B. (A, B) Knockdown of TFAP4 (LNA‐) in Huh7 cells and BEL‐7402 cells decreased migration (A) and invasion (B), and overexpression of LAPTM4B (L+AN) increased migration and invasion in transwell assays (**P < 0.01). Cell migration and invasion were rescued in the group with AP4 knockdown and LAPTM4B overexpression (L+A−). The cells were fixed after 24 h of incubation. (C) Several important tumorigenic genes were detected by western blot analysis. (D) p‐Myc (Thr58) expression was detected by western blot analysis. (E) Cell lines with different c‐Myc levels were detected by western blot analysis. (F) c‐Myc was knocked by siRNA at a working concentration 50 nm or inhibited by a 48‐h incubation with the c‐Myc inhibitor (10058‐F4) at 100 and 200 μm. (G) TFAP4‐LAPTM4B‐c‐Myc may form a positive feedback regulation loop.

To confirm whether AP4 could promote cell migration and invasion via LAPTM4B in HCC cells, transwell assays were conducted. Here, LAPTM4B overexpression significantly promoted the migration and invasion of Huh7 and BEL‐7402 cells in transwell assays, whereas AP4 knockdown reversed these effects (Fig. 6A,B).

We investigated the key regulators involved in cell growth, migration and invasion and found that overexpression of LAPTM4B in Huh7 and BEL‐7402 cells upregulated c‐myc, N‐cadherin and vimentin expression but downregulated E‐cadherin, while knockdown of AP4 in Huh7 and BEL‐7402 cells reversed the expression of the above regulators. The rescued group, with LAPTM4B overexpression and AP4 knockdown, expressed the above regulators at levels not different than control (Fig. 6C).

### 3.5 LAPTM4B feedback regulates AP4 via modulating c‐myc phosphorylation

Importantly, AP4 (Fig. 4A) and c‐myc (Fig. 6C) expression were both higher in the LAPTM4B overexpression group than in the NC group, and AP4 has been identified to be a direct transcriptional target of c‐myc (Jung *et al*., 2008). Moreover, previous research has shown that overexpressing LAPTM4B in HCC cells inhibits c‐myc degradation by promoting its phosphorylation at Thr58 (Yang *et al*., 2010). Thus, we hypothesized that AP4 could not only regulate LAPTM4B to affect c‐myc but could also be feedback‐regulated by LAPTM4B via c‐myc. Our results showed that Huh7 cells and BEL‐7402 cells with stable LAPTM4B overexpression exhibited lower levels of p‐Myc (Thr58). Furthermore, Huh7 cells and BEL‐7402 cells with stable AP4 knockdown exhibited higher levels of c‐myc phosphorylation, which were rescued by LAPTM4B overexpression (Fig. 6D). Moreover, we compared c‐myc expression level in different cell lines (Fig. 6E) and knocked down c‐myc expression by siRNA or inhibited its activity with a c‐myc inhibitor (10058‐F4) and found a significant reduction in AP4 and LAPTM4B expression (Fig. 6F). In conclusion, AP4 could promote HCC cell proliferation, migration and invasion via upregulating LAPTM4B gene transcription by binding to the LAPTM4B gene promoter; then, the overexpression of LAPTM4B inhibits phosphorylation of c‐myc resulting in its accumulation, which then upregulates AP4 expression (Fig. 6G).

### 3.6 AP4 changes the chemotherapy sensitivity of HCC via LAPTM4B by activation of a caspase‐dependent pathway and PI3K/AKT pathway

**Figure 7:**
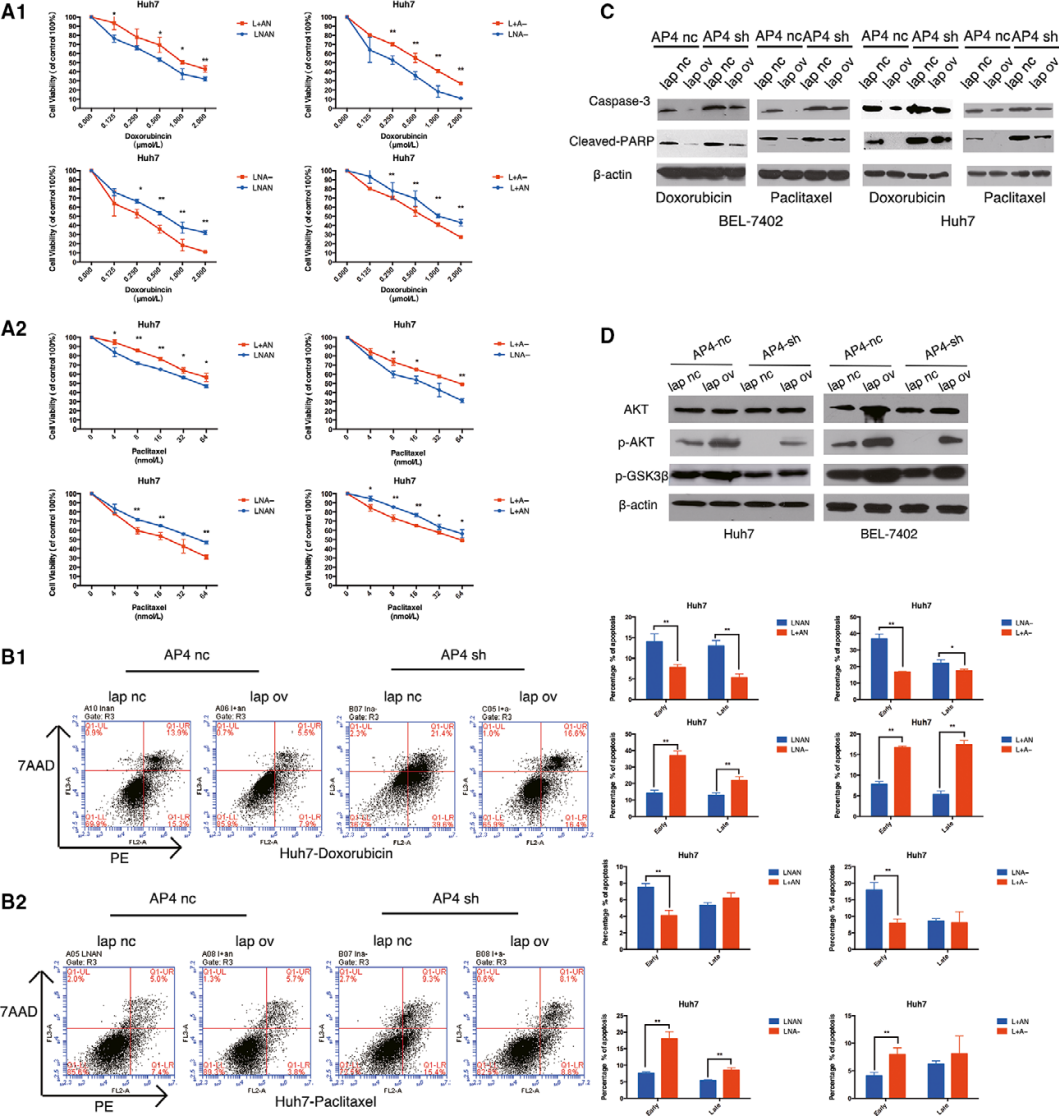
AP4 reduce chemotherapy sensitivity via LAPTM4B by activation of a caspase‐dependent pathway and PI3K/AKT pathway. (A) Viability curves of cells in the presence of various anticancer chemotherapeutic drugs. The LNAN Huh7, L+AN Huh7, LNA‐ Huh7, L+A− Huh7 cells were exposed to increasing concentrations of doxorubicin (A1) or paclitaxel (A2) for 48 h. Cell viabilities were determined by cell viability assay as described in the Materials and methods. The results were expressed as a mean ± SD of viable cell percentage in triplicates from three independent experiments (*P < 0.05, **P < 0.01; Student' *t*‐test). (B) Flow cytometry analysis of apoptosis by antigen‐presenting cell (APC) and 7‐amino‐actinomycin D (7AAD) staining. The LNAN Huh7, L+AN Huh7, LNA‐ Huh7, L+A− Huh7 cells were incubated with 0.25 μmol·L^−1^ doxorubicin (B1) or 8 nmol·L^−1^ paclitaxel (B2). After 48 h of incubation, the cells were harvested and analysed by flow cytometry. Column diagrams of apoptotic cells in percentage (*P < 0.05; **P < 0.01). (C) Protein expression of caspase‐3 and cleaved nuclear poly (ADP‐ribose) polymerase (PARP) was enhanced in the LNA‐ Huh7 cells and LNA‐ BEL‐7402 cells but inhibited in the L+AN Huh7 cells and L+AN BEL‐7402 cells. The protein level of cells with LAPTM4B overexpression and AP4 knockdown (L+A−) was not different from that in control cells, indicating that restoring LAPTM4B expression reversed the effect of AP4 on apoptosis sensitivity. (D) The protein level of p‐AKT, p‐GSK3β and AKT in Huh7 cells and BEL‐7402 cells.

To explore the effects of LAPTM4B on drug sensitivity modulated by AP4, we first examined the viability of Huh7 cells and BEL‐7402 cells expressing high levels of LAPTM4B or low levels of AP4 in a panel of two chemotherapeutic agents with different structures, properties and known mechanisms of action. Compared with control cells, the L+AN Huh7 cells showed a higher viability curve, indicating significantly higher resistance to doxorubicin and paclitaxel. In contrast, LNA− Huh7 cells showed a lower viability curve, indicating significantly lower sensitivity to these two chemotherapeutic agents. Notably, AP4‐shRNA cells with LAPTM4B overexpression (L+A−) exhibited normalized sensitivity to these agents (Fig. 7A1,A2). Similar results were also obtained from BEL‐7402 cell lines (Fig. S4A1,A2).

Moreover, the cellular apoptotic rate was appraised by flow cytometry. As shown in Fig. 5B, the L+AN Huh7 cells were resistant to apoptosis induced by doxorubicin (Fig. 7B1, second) and paclitaxel (Fig. 7B2, second). However, LNA− Huh7 cells were more sensitive to apoptosis induced by doxorubicin (Fig. 7B1, third) and paclitaxel (Fig. 7B2, third). Remarkably, the Huh7 cells with LAPTM4B overexpression and AP4 knockdown exhibited a normalized sensitivity to apoptosis induced by these agents (Fig. 7B1,2, fourth). Similar results were also shown in the BEL‐7402 cell lines (Fig. S5A1,A2). The different sensitivity to apoptosis in the presence of the various chemotherapeutic drugs was further confirmed by analysing the expression of the apoptosis‐related proteins by western blot (Fig. 7C). These results indicate that AP4 inhibits caspase‐dependent apoptosis induced by various chemotherapeutic drugs via increasing the expression of LAPTM4B, providing further evidence that AP4 may change the chemotherapy sensitivity of HCC by inhibiting drug‐induced apoptosis via a caspase‐dependent pathway.

In addition, the PI3K/AKT signalling pathway, the most important mechanism for maintaining cell survival, is also responsible for chemotherapy sensitivity (Abdul‐Ghani *et al*., 2006; Knuefermann *et al*., 2003; Tazzari *et al*., 2007). Upregulated LAPTM4B‐35 could reduce chemotherapy sensitivity by activating PI3K/AKT (Li *et al*., 2010). Thus, we investigated the protein level of p‐AKT, p‐GSK3β and AKT by western blot. As shown in Fig. 7D, the p‐AKT and p‐GSK3β protein expression was enhanced in L+AN cells but inhibited in the LNA− cells. However, the expression in cells with LAPTM4B overexpression and AP4 knockdown (L+A−) was not different than that in control cells, indicating that restoring LAPTM4B expression reversed the effect of AP4 knockdown on multidrug sensitivity.

### 3.7 AP4 and LAPTM4B coexpression in hepatocellular tumours and correlation with HCC prognosis

**Figure 8:**
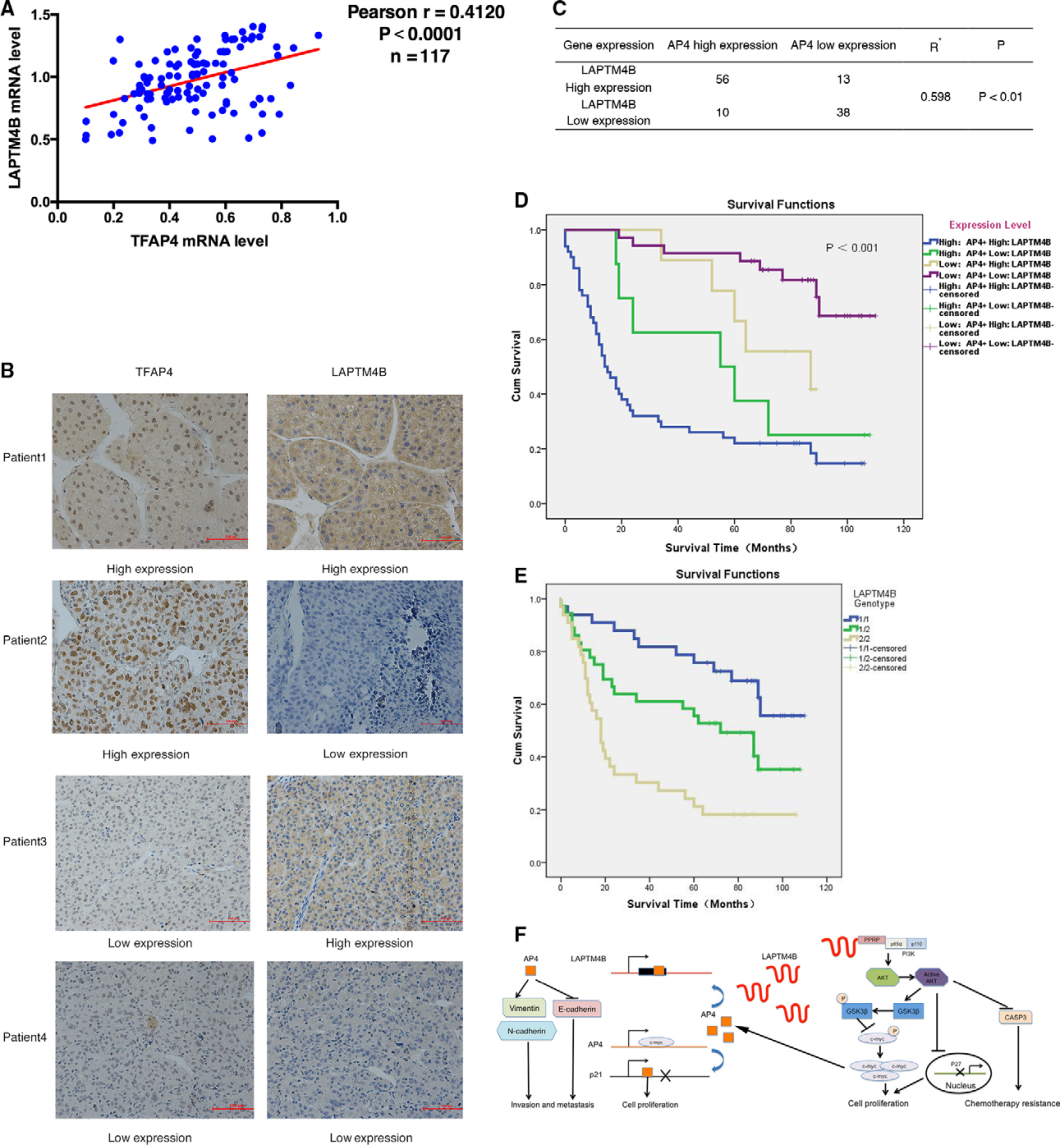
Coexpression of AP4 and LAPTM4B in hepatocellular tumours and correlation with HCC prognosis. (A) AP4 mRNA expression significantly correlated with LAPTM4B mRNA expression in 117 hepatoma cell carcinoma patients. (B, C) One hundred and seventeen primary HCC specimens were analysed by immunohistochemical staining. Original magnification, ×200; scale bar, 100 μm. (D) Survival analysis showed that the cumulative overall survival time ranging from low to high was as follows: high AP4 + high LAPTM4B < high AP4 + low LAPTM4B < low AP4 + high LAPTM4B < low AP4 + low LAPTM4B. (E) Survival analysis showed that the cumulative overall survival time ranging from low to high was as follows: LAPTM4B 2/2 genotype < LAPTM4B 1/2 genotype < LAPTM4B 1/1 genotype. (F) The AP4‐MYC axis could affect tumour growth, invasion and migration as well as multidrug resistance via LAPTM4B in HCC cells.

Finally, we investigated the expression of AP4 and LAPTM4B in patients with hepatocellular tumours. At first, we analysed the expression of AP4 and LAPTM4B mRNA in 117 HCC patients and found that LAPTM4B expression significantly correlated with AP4 expression in HCC specimens (Fig. 8A). The data of TCGA (The Cancer Genome Atlas) database not only showed the same relationship between AP4 and LAPTM4B in mRNA level (Fig. S6A), but also suggested that LAPTM4B expression was associated with tumour grade and high‐expression LAPTM4B was a poor prognostic marker in HCC patients (Fig. S6B). Next, we performed immunohistochemical staining using the anti‐AP4 antibody and anti‐LAPTM4B antibody and found a statistically significant positive correlation between AP4 and LAPTM4B expression in HCC (Fig. 8B,C). Survival analysis ranked the cumulative overall survival time as follows: high AP4 + high LAPTM4B < high AP4 + low LAPTM4B < low AP4 + high LAPTM4B < low AP4 + low LAPTM4B in HCC. Thus, high expression of AP4 together with high expression of LAPTM4B was associated with poor prognosis for HCC patients. Inversely, patients with low expression of AP4 and low expression of LAPTM4B had the longest survival time (Fig. 8D). Considering the LAPTM4B genotype as the marker of prognosis, we could find that cumulative overall survival time was as follows: LAPTM4B 2/2 genotype < LAPTM4B 1/2 genotype < LAPTM4B 1/1 genotype. Therefore, we can suggest that LAPTM4B 2/2 genotype may combine more AP4 protein in LAPTM4B promoter region so that promote LAPTM4B function in HCC leading to a poor prognosis.

## 4 Discussion

In this study, we first screened for transcription factors that bind to the 19‐bp polymorphism sequence of the LAPTM4B promoter region and found that AP4 could promote HCC tumour growth, metastasis but reduce chemotherapy sensitivity by positively regulating LAPTM4B expression. Moreover, we examined the relationship between c‐myc, AP4 and LAPTM4B and found a c‐myc‐AP4‐LAPTM4B‐positive feedback loop that may also be a pivotal factor in HCC tumour survival. Finally, we showed that AP4 and LAPTM4B were highly coexpressed in HCC tissues, and their coexpression might be a marker of poor prognosis in HCC patients.

Previous studies have shown that LAPTM4B allele*2 was a susceptibility factor for various cancers; however, the reason for this susceptibility is still unknown. Owing to the location of the polymorphism in the 5ʹ‐UTR region, we hypothesized that unique transcription factors that bind to this polymorphism region were the key regulators of this tumour susceptibility.

To explore the mechanism behind this tumour susceptibility, we first investigated the transcription factors that bind the region of the LAPTM4B promoter polymorphism. We found that only AP4 had binding sites within the 19‐bp polymorphism region both in the LAPTM4B*1 promoter and LAPTM4B*2 promoter. Although DNA pull‐down and subsequent mass spectrometry assay were not performed to validate transcription factors furthermore, we still found AP4, the special transcription factor within the 19‐bp sequence by bioinformatics prediction and TF activation profiling plate array assays. Luciferase reporter assay results showed that mutation of both binding sites in the LAPTM4B*2 promoter resulted in the lowest transcriptional activity, suggesting that AP4 has a much greater effect on transcription of LAPTM4B allele*2. Furthermore, the ChIP‐qPCR assay suggested that more AP4 bound the LAPTM4B allele*2 *in vivo*, which further demonstrates that AP4 plays a key role in LAPTM4B transcription regulation.

Previous studies have proven that AP4 could be a positive or negative regulator of tumour‐related genes by binding the E‐box of their promoters (Jackstadt *et al*., 2013; Liu *et al*., 2017). Here, we demonstrated that AP4 could bind within the LAPTM4B polymorphism region *in vitro* and *in vivo* and function as a positive transcriptional regulator. Moreover, we knocked down AP4 using three siRNA and one shRNA and found that the protein level and mRNA level of LAPTM4B decreased along with those of AP4, further demonstrating that AP4 could positively regulate LAPTM4B expression not only at the transcriptional level but also at the protein level.

To investigate the effect of AP4 on LAPTM4B function in HCC, cell proliferation and tumour growth conditions were first examined. LAPTM4B has been shown to boost tumour growth and cell proliferation by activating related signalling pathways in various kinds of tumours (Kadara *et al*., 2014; Zhou *et al*., 2010). However, AP4, as a transcription factor, has been demonstrated to be a double‐edged sword in tumour growth and cell proliferation. On the one hand, AP4 has been shown to directly inhibit p21 and p16 to suppress cell senescence (Jackstadt *et al*., 2013). On the other hand, AP4 interacts with other transcription factors to promote expression of some negative regulators of cell growth, such as Src homology region 2 domain‐containing phosphatase‐1 (SHP1), in breast cancer cells (Amin *et al*., 2011). Our results suggested that AP4 could increase HCC cell proliferation and tumour growth via LAPTM4B *in vitro* or *in vivo*. In keeping with the previous results, fluorescence‐activated cell sorting analysis of the effects of AP4 knockdown on the cell cycle in HCC cells revealed a concomitant increase in cells in G0/G1, which was also rescued by restoration of LAPTM4B expression, thereby relieving the cell of the proliferation inhibition. Immunoblot analysis of negative cell cycle regulators indicated that the G0/G1 arrest by AP4 knockdown was most likely associated with the induction of p21 and p27, major players in G1 arrest, and the reduction in cyclin E, the cell cycle promoter. In this regard, AP4 promotes HCC cell proliferation not only by suppressing cell cycle inhibitors directly but also via positive regulation of oncogene LAPTM4B.

The effect of AP4 via LAPTM4B on the migration and invasion of HCC cells was then analysed. In this research, transwell assays showed that the inhibition of migration and invasion induced by AP4 knockdown could be abrogated by restoring LAPTM4B expression, which was consistent with the expression of epithelial–mesenchymal transition (EMT)‐related proteins on immunoblot analysis. Although a series of clinical experiments have shown that various kinds of carcinomas with LAPTM4B‐35 overexpression present more invasive characteristics, the mechanism of the effect of LAPTM4B on migration and invasion is still unclear (Xiao *et al*., 2017). Previous researches suggested that the possible mechanism of the effect of LAPTM4B on migration and invasion was the interaction between LAPTM4B and SH3 domain‐containing proteins that are involved in many signalling pathways (Liu *et al*., 2009). In this study, the higher expression of AP4 and c‐myc in LAPTM4B‐overexpressing Huh7 and BEL‐7402 cells attracted our attention. Importantly, c‐myc is a positive transcription factor of AP4 (Jung *et al*., 2008), and overexpression of LAPTM4B increases the level of c‐myc by inhibiting its phosphorylation at Thr58 (Yang *et al*., 2010). On the basis of immunoblot analysis of p‐myc, we suggested that AP4 could affect phosphorylation of c‐myc via LAPTM4B. By interfering with c‐myc function via siRNA or a c‐myc inhibitor, we demonstrated that c‐myc could influence LAPTM4B expression via AP4. In conclusion, LAPTM4B may regulate AP4 expression via a positive feedback loop involving EMT activation by c‐myc accumulation, which could promote migration, invasion and proliferation (Jackstadt *et al*., 2013).

In addition to cell proliferation, migration and invasion, AP4 may also affect chemotherapy sensitivity via LAPTM4B in HCC. We observed that AP4 knockdown made the Huh7 cells and BEL‐7402 cells more sensitive to paclitaxel and doxorubicin, and this sensitivity was rescued by restoration of LAPTM4B expression. On the one hand, AP4 could lead to chemotherapy agent sensitivity depression via LAPTM4B by modulating the caspase apoptosis pathway; on the other hand, AP4 could regulate LAPTM4B to activate PI3K/AKT signalling and inactivate GSK3β, leading to c‐myc accumulation and amplification of the effect of the PI3K/AKT pathway on drug resistance. In addition, regulation of LAPTM4B by AP4 may depress chemotherapy sensitivity through AKT signalling activation by blocking epidermal growth factor receptor (EGFR) degradation (Tan *et al*., 2015) or enhancing the efflux of chemotherapy agents with the aid of P‐glycoprotein (Li *et al*., 2010). Although previous studies have shown that AP4 could play a positive (Liu *et al*., 2012) or negative role (Buechler, 2009) in chemotherapy sensitivity in different carcinomas, we have shown that AP4 depresses the chemotherapy sensitivity of HCC via LAPTM4B in this research.

As AP4 could promote cell proliferation and metastasis but reduce chemotherapy sensitivity via LAPTM4B, we examined whether coexpression of AP4 and LAPTM4B could be treated as a prognostic marker in HCC patients. Here, we found that expression levels of AP4 and LAPTM4B were positively correlated at both the mRNA and protein levels. Moreover, high expression of AP4 and LAPTM4B together was a marker of poor prognosis in HCC patients.

In this research, AP4 has been demonstrated to bind to the polymorphism region of LAPTM4B promoter and be the key transcriptional regulator of LAPTM4B. In the future study, we will focus on the functional difference of AP4 in LAPTM4B*1 allele one and LAPTM4B*2 allele two and explore the cofactors in this polymorphism region.

## 5 Conclusion

In this research, we first reported the significance of the AP4‐LAPTM4B axis in mediating HCC pathogenesis and chemodrug sensitivity. Interestingly, LAPTM4B was shown to also regulate AP4 expression via its effects on c‐myc, resulting in a circular positive feedback loop. Although some research has shown that c‐myc works together with AP4 to regulate tumour pathogenesis (Xue *et al*., 2016) or that AP4 regulates the Wnt signalling pathway (Lebensohn *et al*., 2016), which also involves c‐myc, our report is the first to show that AP4 also regulates c‐myc by affecting LAPTM4B expression. As summarized in Fig. 8F, AP4 positively regulates LAPTM4B to promote cell growth and metastasis but reduce chemotherapy sensitivity; additionally, LAPTM4B positively regulates AP4 expression via c‐myc to further amplify the positive effect of AP4 and LAPTM4B on HCC. These interactions between AP4 and LAPTM4B may explain the clinical results indicating that HCC patients with a high expression of AP4 and LAPTM4B had a poor prognosis. In this regard, we can conclude that the AP4‐LAPTM4B axis plays a pivotal role in HCC pathogenesis and chemodrug sensitivity.

## Author contributions

YM and LW designed and performed experiments. YM wrote the manuscript. JX analysed the data. QZ supervised the experiments.

## Supporting information


**Fig. S1.** The result of TF activation profiling plate assay.
**Fig. S2.** Genotyping of LAPTM4B. M is DNA marker.
**Fig. S3.** AP4 promotes HCC cell growth via LAPTM4B by affecting cell proliferation and cell cycle *in vitro* and *in vivo*.
**Fig. S4.** AP4 reduce chemotherapy sensitivity via LAPTM4B.
**Fig. S5.** (A) Flow cytometry analysis of apoptosis by APC and 7AAD staining.
**Fig. S6.** TCGA dataset information about 373 HCC patients.
**Fig. S7.** All the plasmids digested by Xho1 and Hind3 enzyme.
**Fig. S8.** The sequenced results of mutation plasmids.
**Fig. S9.** Eleven kinds of plasmids transfected into cells.

**Table S1.** LAPTM4B*1 allele transcription factor prediction results of online database.
**Table S2.** LAPTM4B*2 allele transcription factor prediction results of online database.
**Table S3.** Primers for luciferase plasmids construction.
**Table S4.** The siRNA target sequences of AP4.
**Table S5.** The primer of AP4, LAPTM4B and GAPDH.
**Table S6.** Antibodies used in WB.
**Table S7.** Relationship between AP4 expression and clinicopathological features of HCC.
**Table S8.** Relationship between LAPTM4B‐35 expression and clinicopathological features of HCC.
