# Supplementary material for: AP4 positively regulates LAPTM4B to promote hepatocellular carcinoma growth and metastasis, while reducing chemotherapy sensitivity
Source: Mol Oncol. 2018 Feb 6;12(3):373–90. doi: 10.1002/1878-0261.12171 (PMC5830630; doi:10.1002/1878-0261.12171)
Supplement: Supplementary file 1 — Fig. S1. The result of TF activation profiling plate assay. Fig. S2. Genotyping of LAPTM4B. M is DNA marker. Fig. S3. AP4 promotes HCC cell growth via LAPTM4B by affecting cell proliferation and cell cycle in vitro and in vivo. Fig. S4. AP4 reduce chemotherapy sensitivity via LAPTM4B. Fig. S5. (A) Flow cytometry analysis of apoptosis by APC and 7AAD staining. Fig. S6. TCGA dataset information about 373 HCC patients. Fig. S7. All the plasmids digested by Xho1 and Hind3 enzyme. Fig. S8. The sequenced results of mutation plasmids. Fig. S9. Eleven kinds of plasmids transfected into cells. [file MOL2-12-373-s001.pdf]

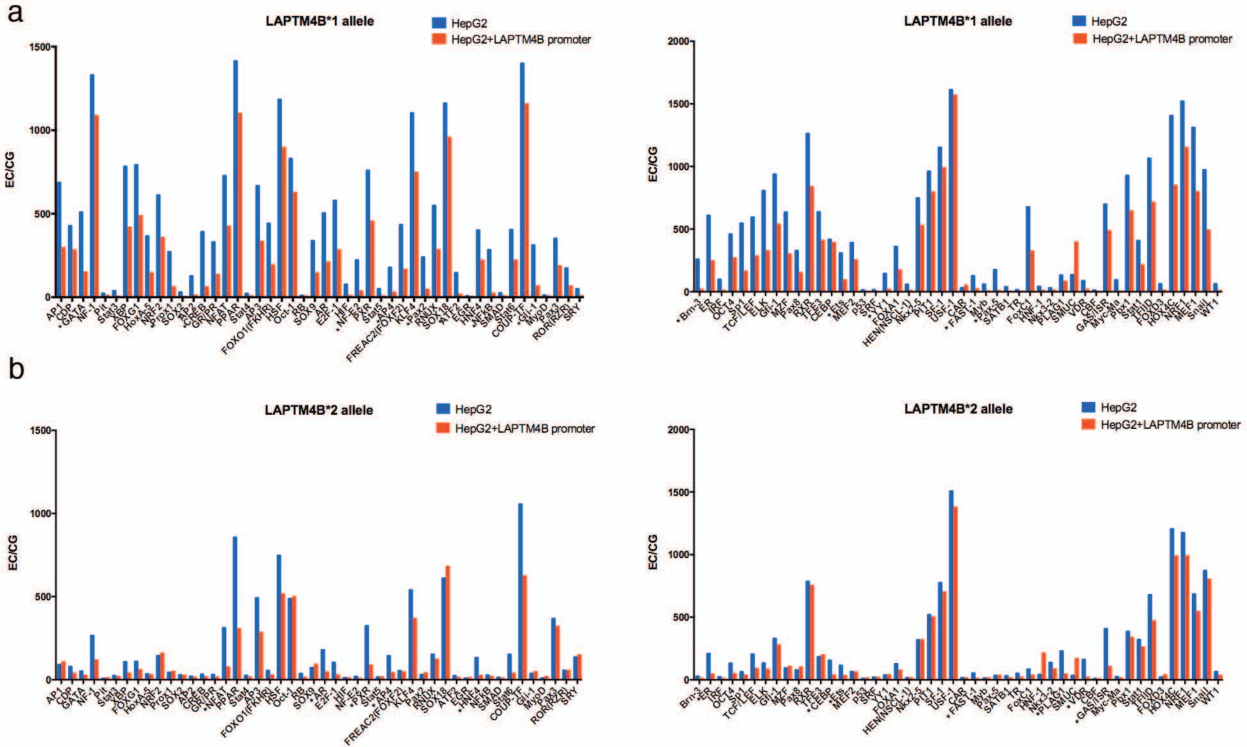
a
LAPTM4B*1 allele
HepG2
HepG2+LAPTM4B promoter
EC/CG
LAPTM4B*1 allele
HepG2
HepG2+LAPTM4B promoter
EC/CG
b
LAPTM4B*2 allele
HepG2
HepG2+LAPTM4B promoter
EC/CG
LAPTM4B*2 allele
HepG2
HepG2+LAPTM4B promoter
EC/CG

FigureS1. The result of TF activation profiling plate assay. (a) The result of TF activation profiling plate assay in LAPTM4B*1 promoter. (b) The result of TF activation profiling plate assay in LAPTM4B*1 promoter. The factors with asterisk represent binding factors，which means the HepG2 nuclear protein+LAPTM4B promoter group compete more than 3-folds protein than HepG2 nuclear protein group and each factor's value of chemiluminescence must be above 100.

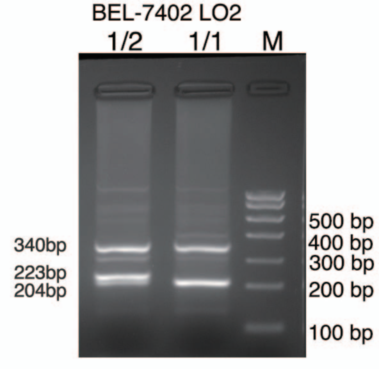
BEL-7402 LO2
1/2
1/1
M
340bp
223bp
204bp
500 bp
400 bp
300 bp
200 bp
100 bp

FigureS2. Genotyping of LAPTM4B. M is DNA marker. LO2 gennotype is LAPTM4B*1/1. BEL-7402 genotype is LAPTM4B*1/2. 204bp is the LAPTM4B*1 allele sequence, 223bp is the LAPTM4B*2 allele sequence, 340bp is the β-actin sequence.

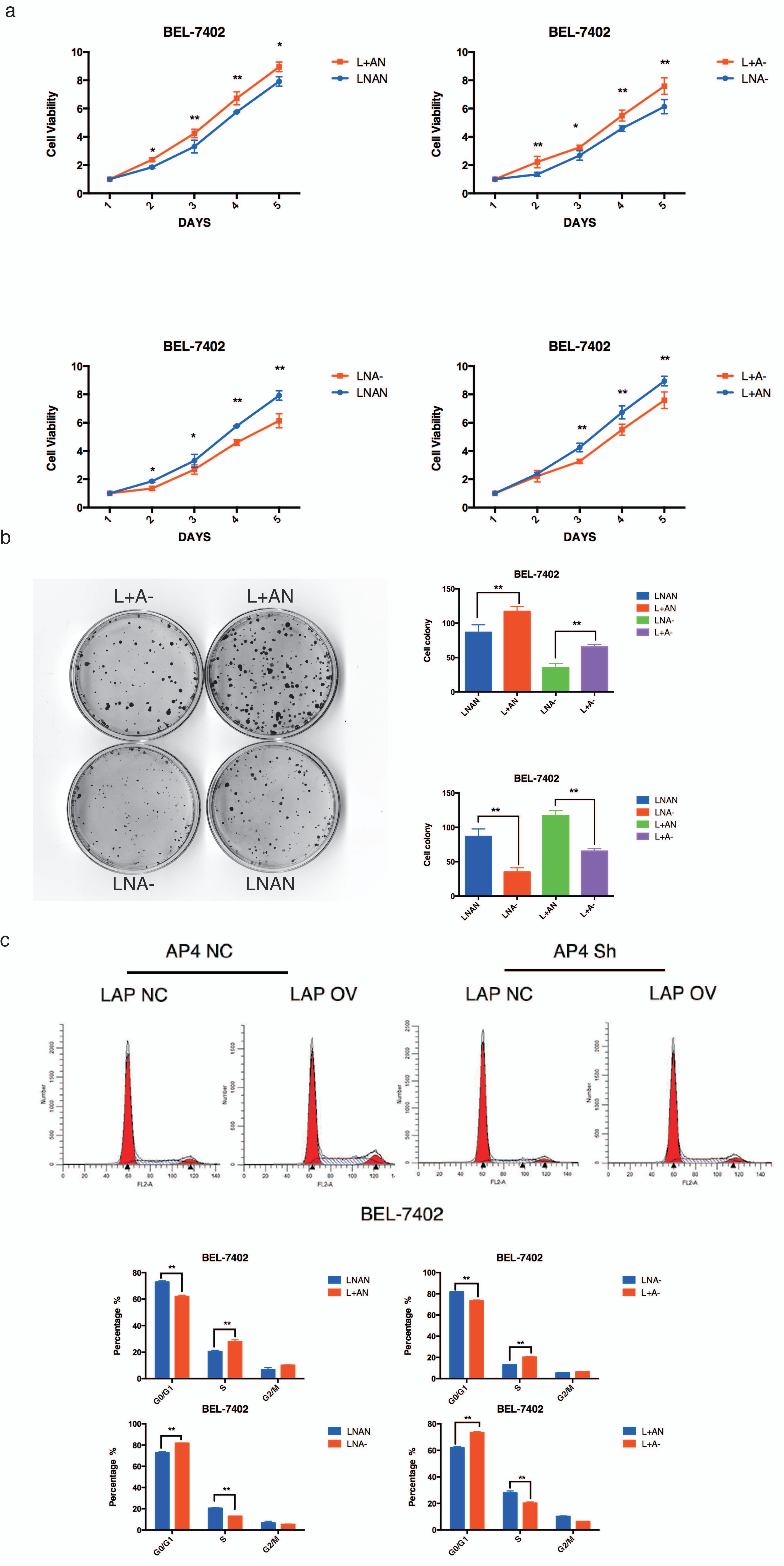
a
BEL-7402
Cell Viability
DAYS
L+AN
LNAN
L+A-
LNA-
b
L+A-
L+AN
LNA-
LNAN
Cell colony
c
AP4 NC
AP4 Sh
LAP NC
LAP OV
Number
FL2-A
BEL-7402
Percentage %
G0/G1
S
G2/M

FigureS3. AP4 promotes HCC cell growth via LAPTM4B by affecting cell proliferation and cell cycle *in vitro* and *in vivo.* **(a-b)** Overexpression of LAPTM4B partially but significantly rescued the cell growth arrest induced by TFAP4 knockdown in BEL-7402 cells, as measured by the cell viability assay and colony formation (*P<0.05, ** P<0.01). **(c)** Restoration of LAPTM4B significantly reversed the cycle arrest at G1 phase induced by AP4 knockdown in BEL-7402 cells. (n=3, mean ± s.d.).

a1

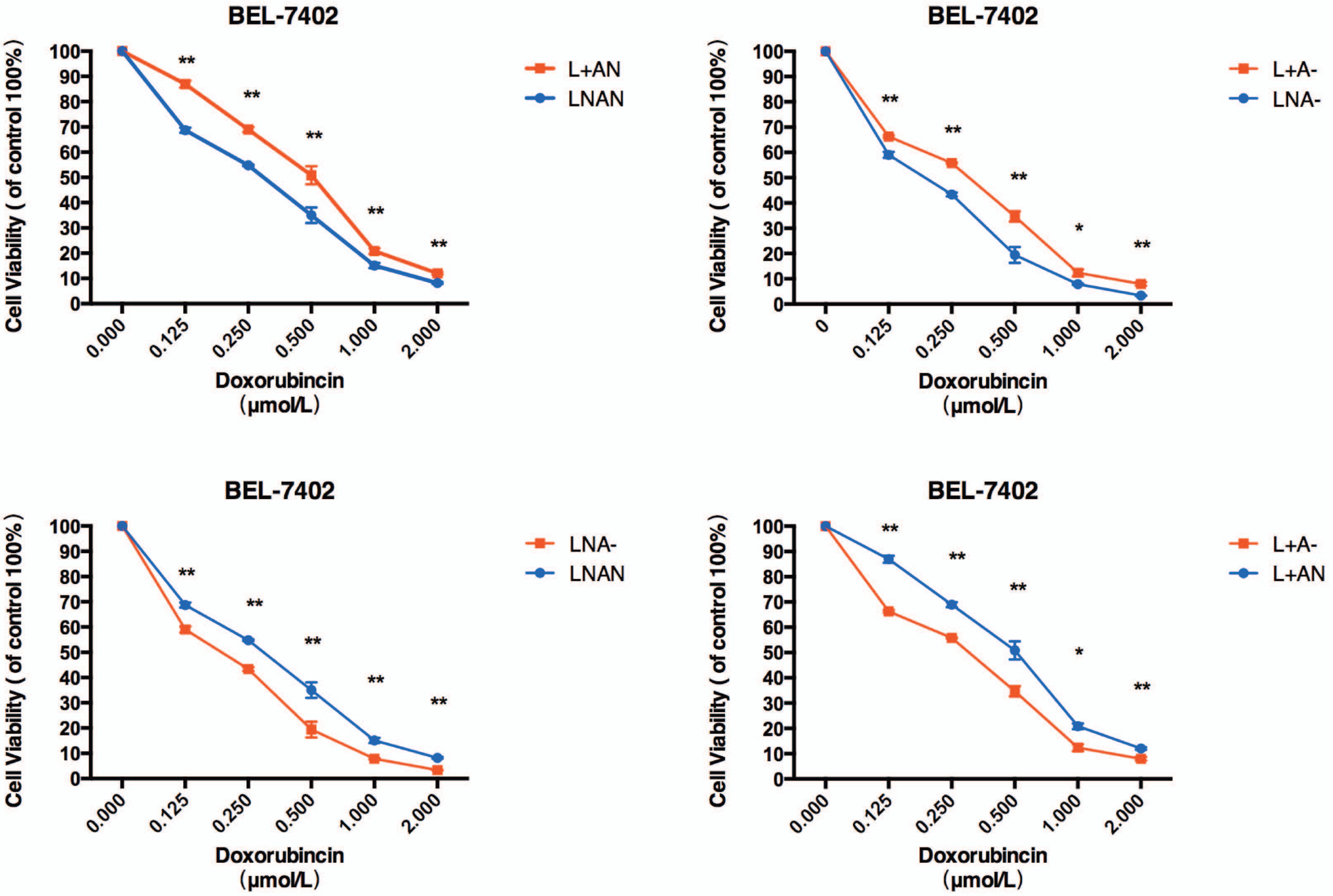
BEL-7402
Cell Viability (of control 100%)
L+AN
LNAN
Doxorubincin
(μmol/L)
0.000
0.125
0.250
0.500
1.000
2.000
BEL-7402
L+A-
LNA-
BEL-7402
LNA-
LNAN
BEL-7402
L+A-
L+AN

a2

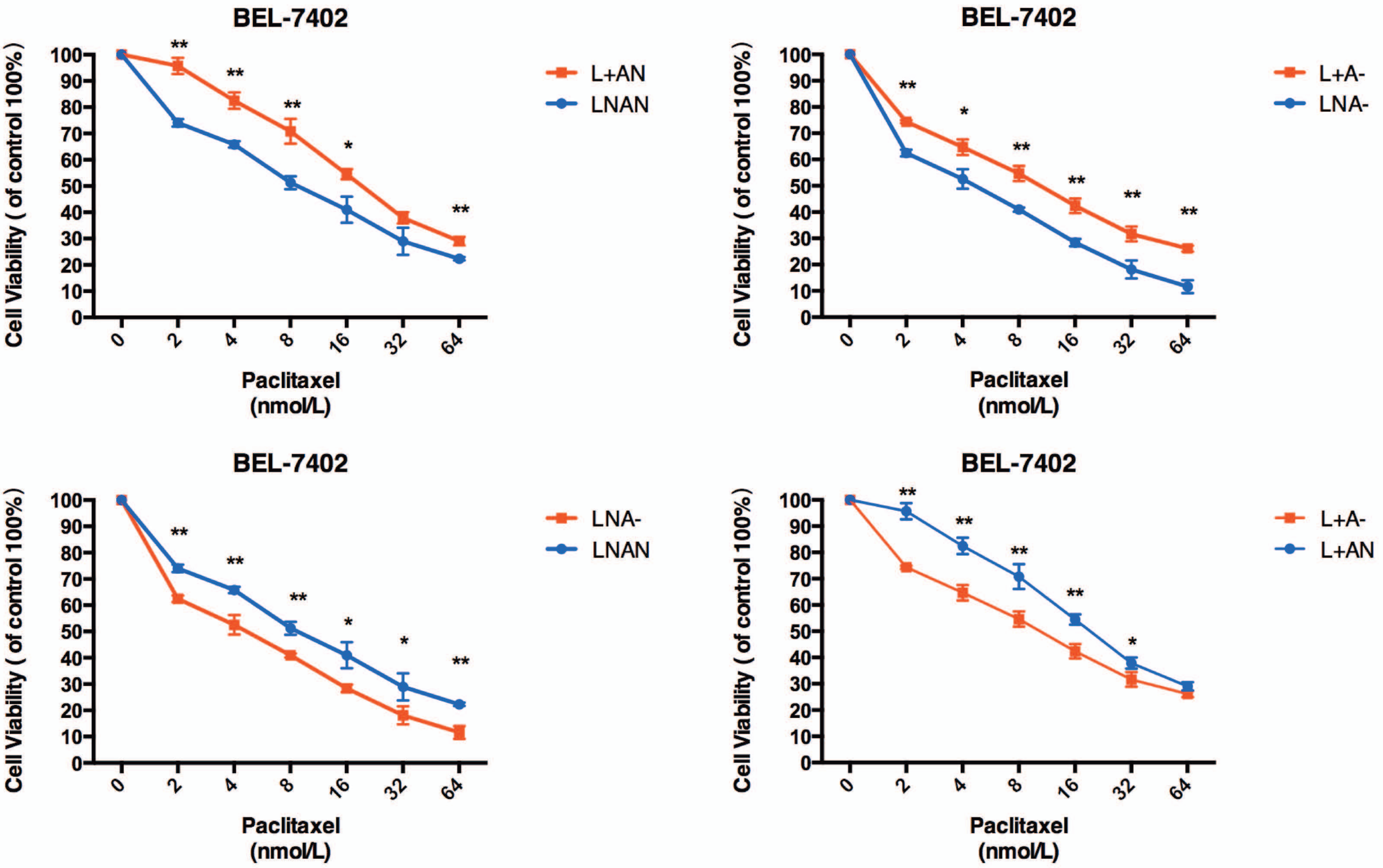
BEL-7402
Cell Viability (of control 100%)
L+AN
LNAN
Paclitaxel
(nmol/L)
0
2
4
8
16
32
64
BEL-7402
L+A-
LNA-
BEL-7402
LNA-
LNAN
BEL-7402
L+A-
L+AN

FigureS4. AP4 reduce chemotherapy sensitivity via LAPTM4B. **(a)** Viability curves of cells in the presence of various anticancer chemotherapeutic drugs. The LNAN BEL-7402, L+AN BEL-7402, LNA- BEL-7402, L+A-BEL-7402 cells were exposed to increasing concentrations of doxorubicin **(a1)** or paclitaxel **(a2)** respectively, for 48 h. Cell viabilities were determined by cell viability assay as described in materials and methods. Results were expressed as a mean±s.d. of viable cell percentage in triplicates from three independent experiments. (*P<0.05; **P<0.01, Student' *t*-test).

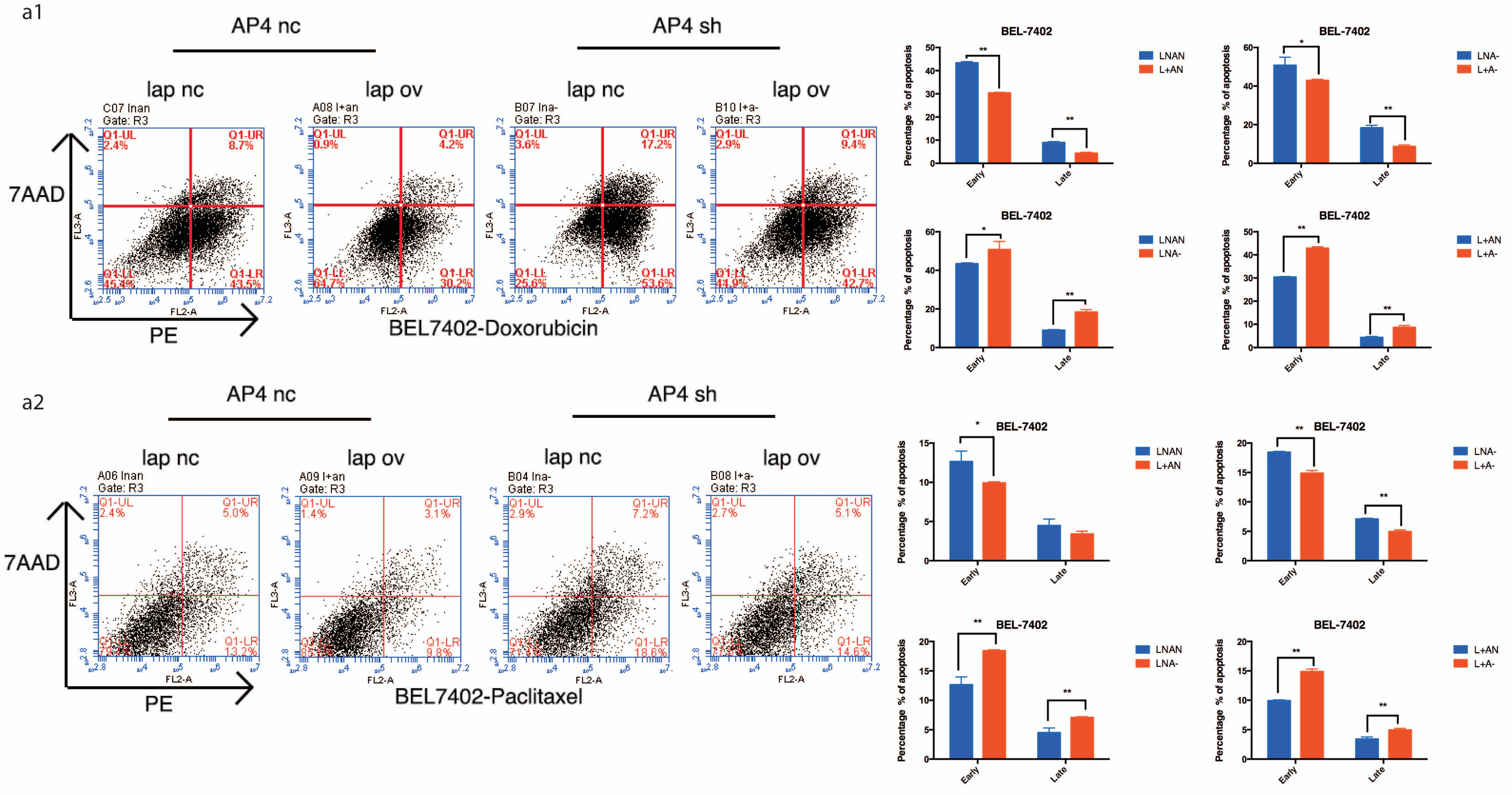

a1
AP4 nc
AP4 sh
lap nc
lap ov
lap nc
lap ov
C07 lnan
Gate: R3
A08 l+an
Gate: R3
B07 lna-
Gate: R3
B10 l+a-
Gate: R3
Q1-UL 2.4%
Q1-UR 8.7%
Q1-LL 45.4%
Q1-LR 43.5%
Q1-UL 0.9%
Q1-UR 4.2%
Q1-LL 64.7%
Q1-LR 30.2%
Q1-UL 3.6%
Q1-UR 17.2%
Q1-LL 25.6%
Q1-LR 53.6%
Q1-UL 2.9%
Q1-UR 9.4%
Q1-LL 44.9%
Q1-LR 42.7%
7AAD
PE
FL3-A
FL2-A
BEL7402-Doxorubicin
BEL-7402
Percentage % of apoptosis
Early
Late
LNAN
L+AN
LNA-
L+A-
a2
AP4 nc
AP4 sh
lap nc
lap ov
lap nc
lap ov
A06 lnan
Gate: R3
A09 l+an
Gate: R3
B04 lna-
Gate: R3
B08 l+a-
Gate: R3
Q1-UL 2.4%
Q1-UR 5.0%
Q1-LR 13.2%
Q1-UL 1.4%
Q1-UR 3.1%
Q1-LR 9.8%
Q1-UL 2.9%
Q1-UR 7.2%
Q1-LR 18.6%
Q1-UL 2.7%
Q1-UR 5.1%
Q1-LR 14.6%
7AAD
PE
FL3-A
FL2-A
BEL7402-Paclitaxel
BEL-7402
Percentage % of apoptosis
Early
Late
LNAN
L+AN
LNA-
L+A-

FigureS5 (a) Flow cytometry analysis of apoptosis by APC and 7AAD staining. The LNAN BEL-7402, L+AN BEL-7402, LNA- BEL-7402, L+A- BEL-7402 cells were incubated with 0.25μmol/l doxorubicin **(a1)**, 8 nmol/l paclitaxel **(a2)** After 48 h incubation, the cells were harvested and analyzed by flow cytometer. Column diagrams of apoptotic cells in percentage. (*$P<0.05$; **$P<0.01$).

a

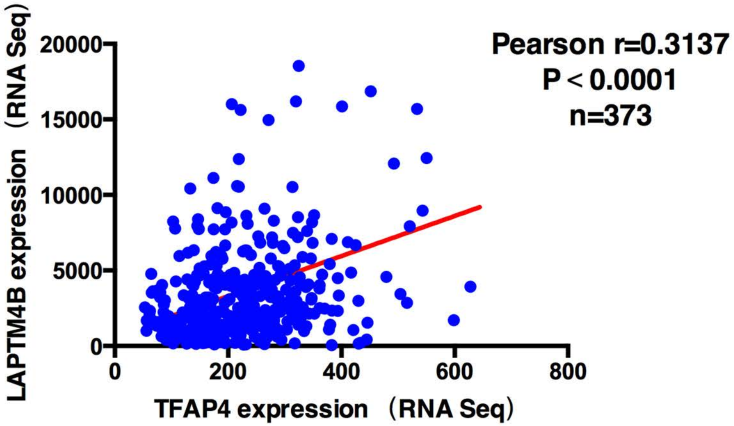
Pearson r=0.3137
P < 0.0001
n=373
LAPTM4B expression （RNA Seq）
TFAP4 expression （RNA Seq）
20000
15000
10000
5000
0
0
200
400
600
800

b

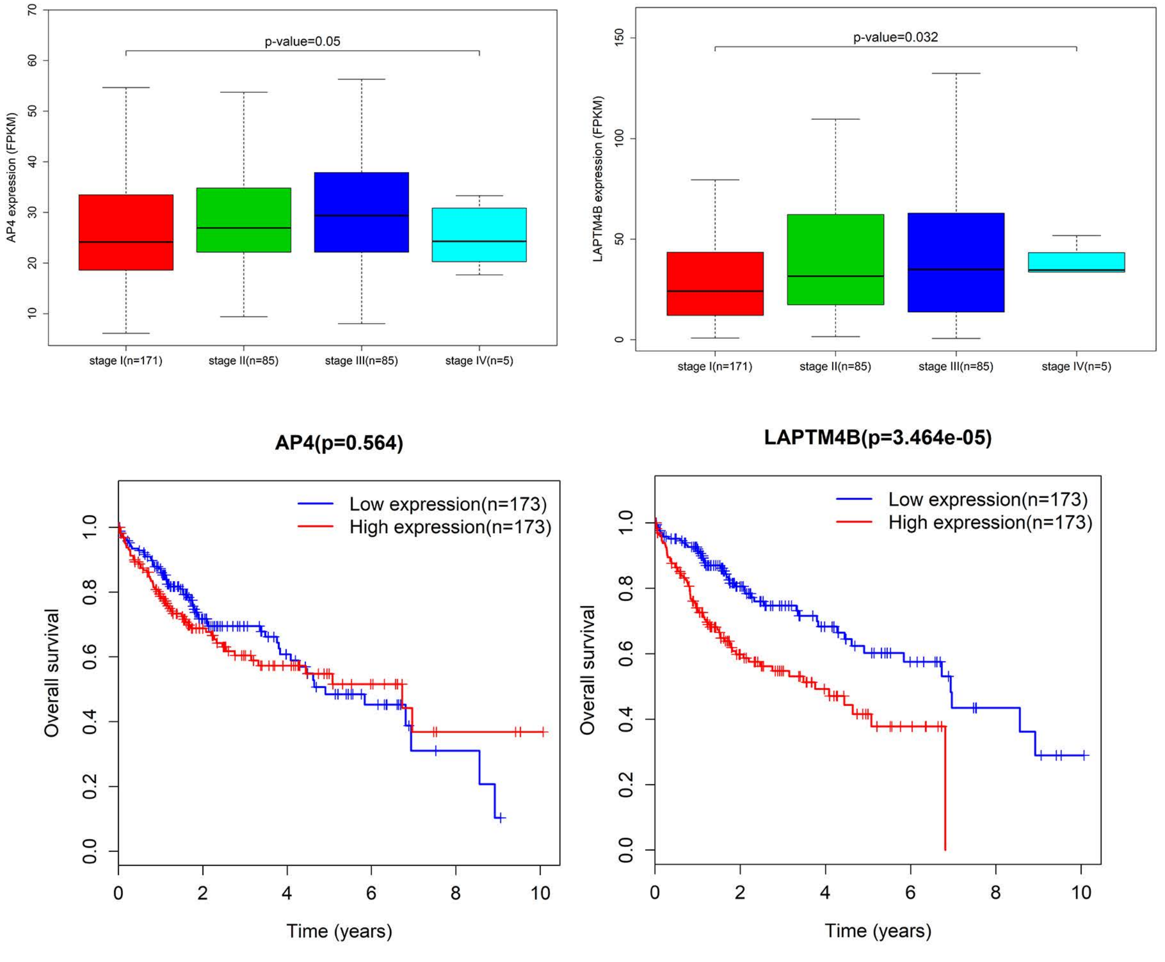
p-value=0.05
AP4 expression (FPKM)
stage I(n=171)
stage II(n=85)
stage III(n=85)
stage IV(n=5)
p-value=0.032
LAPTM4B expression (FPKM)
stage I(n=171)
stage II(n=85)
stage III(n=85)
stage IV(n=5)
AP4(p=0.564)
Low expression(n=173)
High expression(n=173)
Overall survival
Time (years)
LAPTM4B(p=3.464e-05)
Low expression(n=173)
High expression(n=173)
Overall survival
Time (years)

FigureS6. TCGA dataset information about 373 HCC patients. **(a)** AP4 mRNA expression significantly correlated with LAPTM4B mRNA expression in 373 hepatoma cell carcinoma patients from TCGA. **(b-c)** The association of AP4 and LAPTM4B with tumour grades and HCC patients' survival.

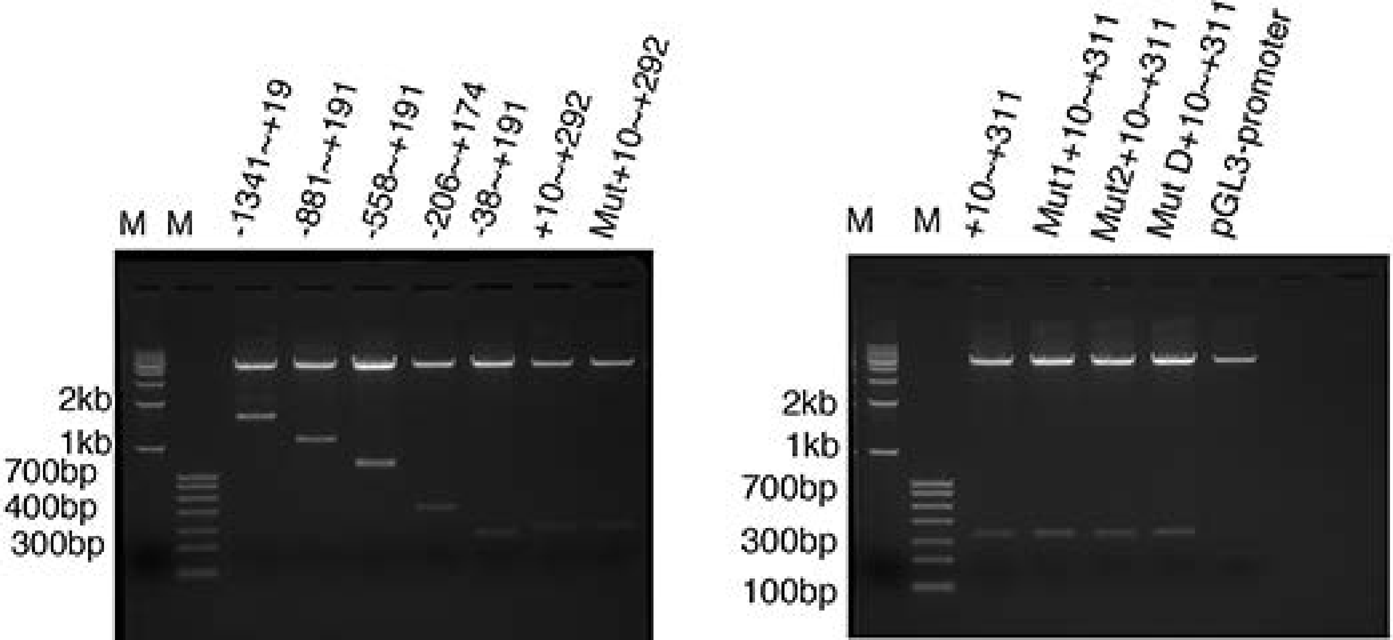

M
M
-1341~+19
-881~+191
-558~+191
-206~+174
-38~+191
+10~+292
Mut+10~+292
2kb
1kb
700bp
400bp
300bp
M
M
+10~+311
Mut1+10~+311
Mut2+10~+311
Mut D+10~+311
pGL3-promoter
2kb
1kb
700bp
300bp
100bp

FigureS7. All the plasmids digested by Xho1 and Hind3 enzyme. Mut+10~+292represents the LAPTM4B allele*1 promoter plasmid mutated the AP4 binding site. Mut1+10~+311 represents the LAPTM4B allele*2 promoter plasmid mutated the first AP4 binding site. Mut2+10~+311 represents the LAPTM4B allele*2 promoter plasmid mutated the second AP4 binding site. Mut D+10~+311 represents the LAPTM4B allele*2 promoter plasmid mutated both AP4 binding sites.

a.

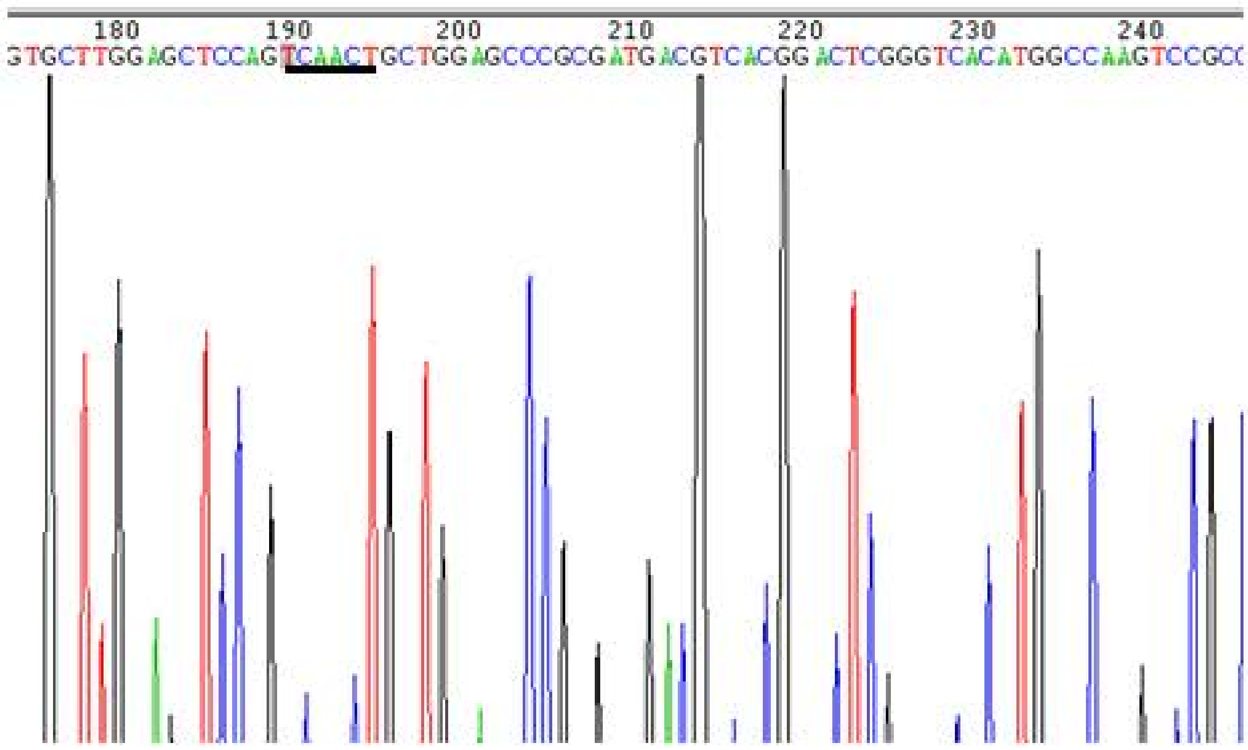
180 190 200 210 220 230 240
GTGCTTGGAGCTCCAGTTCAACTGCTGGAGCCCGCGATGACGTCACGGACTCGGGTCACATGGCCAAGTCCGC

b.

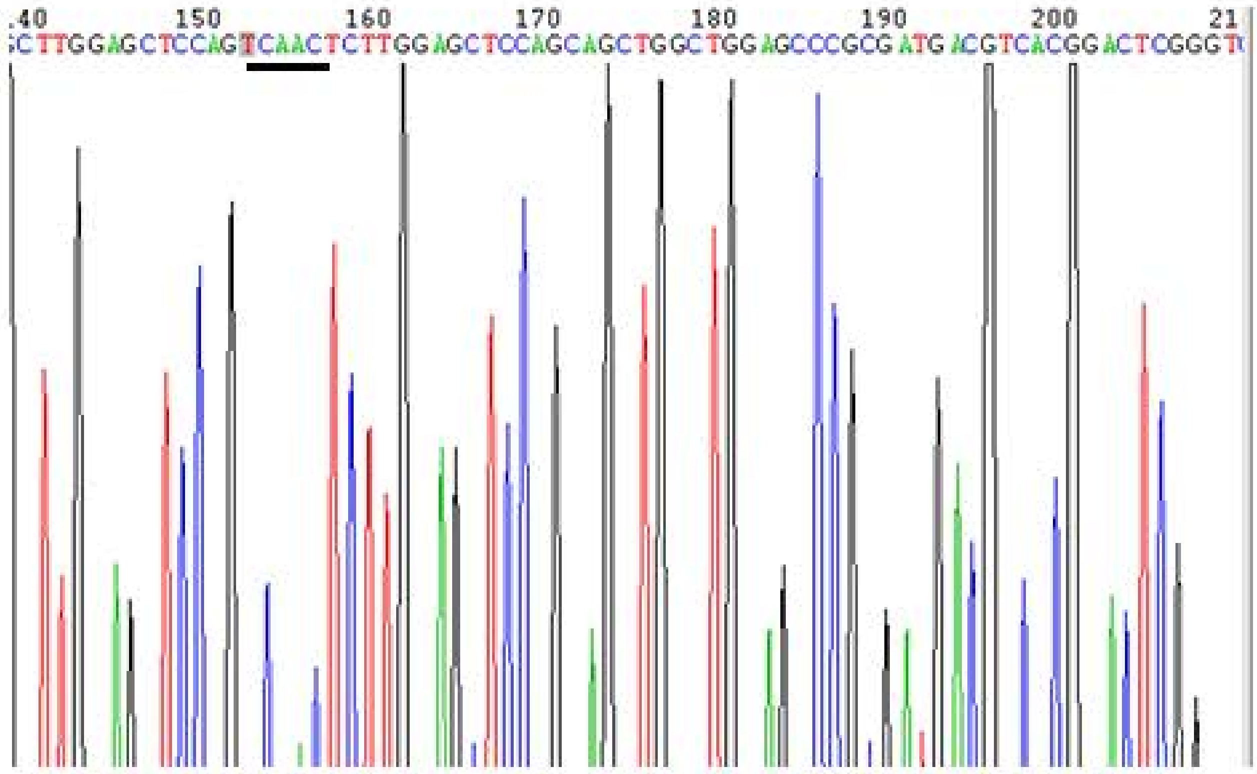
140 150 160 170 180 190 200 21
CTTGGAGCTCCAGTTCAACTCTTGGAGCTCCAGCAGCTGGCTGGAGCCCGCGATGACGTCACGGACTCGGGT

c.

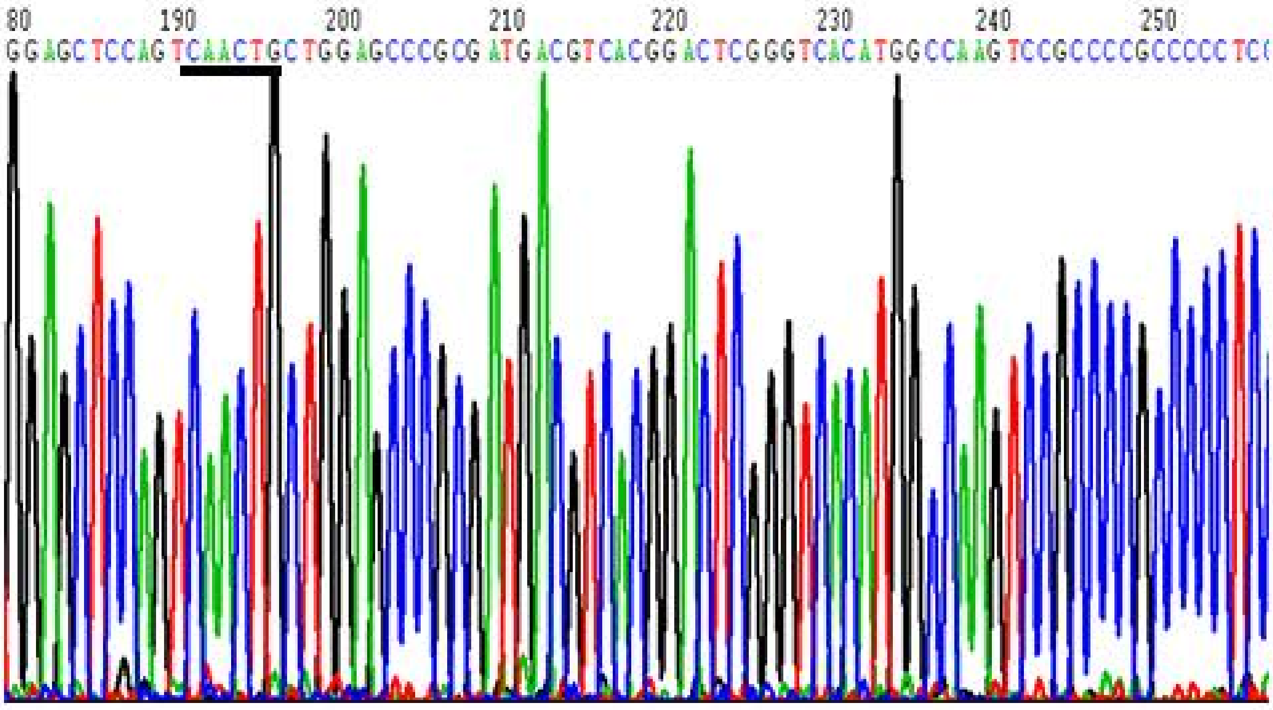
80 190 200 210 220 230 240 250
GGAGCTCCAGTCAACTGCTGGAGCCCGCGATGACGTCACGGACTCGGGTCACATGGCCAAGTCCGCCCCGCCCCCTC

d.

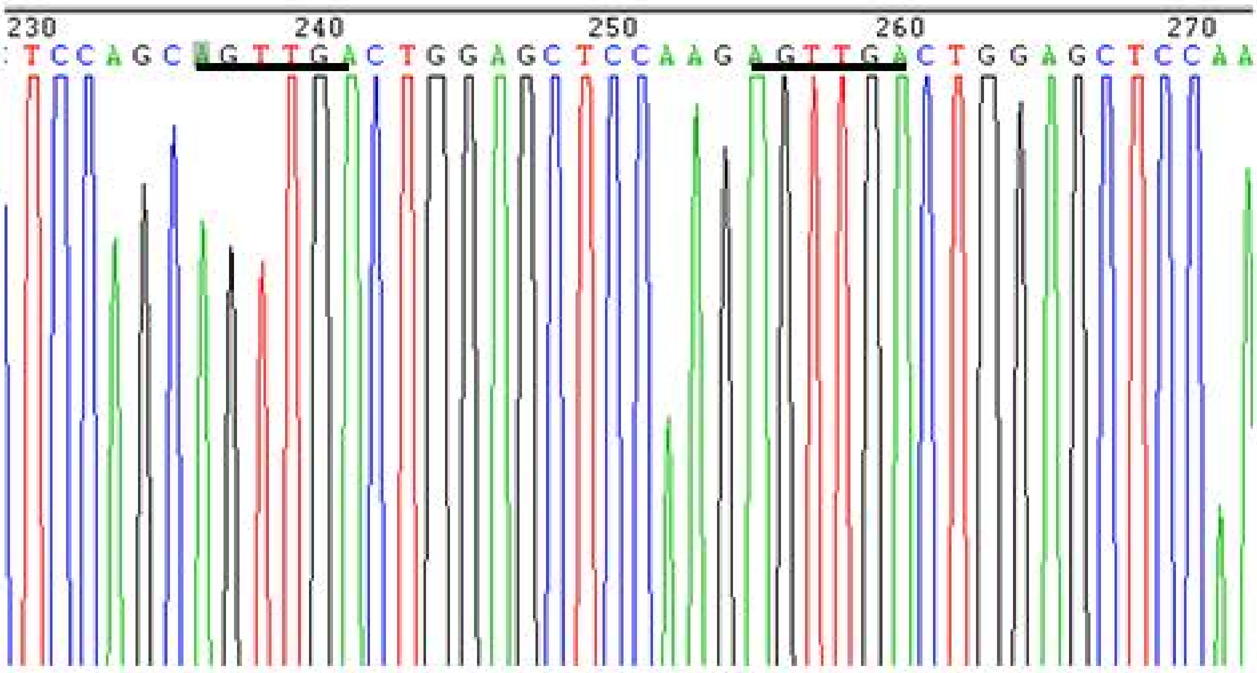
230 240 250 260 270
TCCAGCAGTTGACTGGAGCTCCAAGAGTTGACTGGAGCTCCAA

Figure S8. The sequenced results of mutation plasmids. TCAACT with underline were mutation sequence in **(a)** Mut +10-+292 plasmid, **(b)** Mut1 +10-+311 plasmid, **(c)** Mut2 +10-+311 plasmid and **(d)** Mut D +10-+311 plasmid.

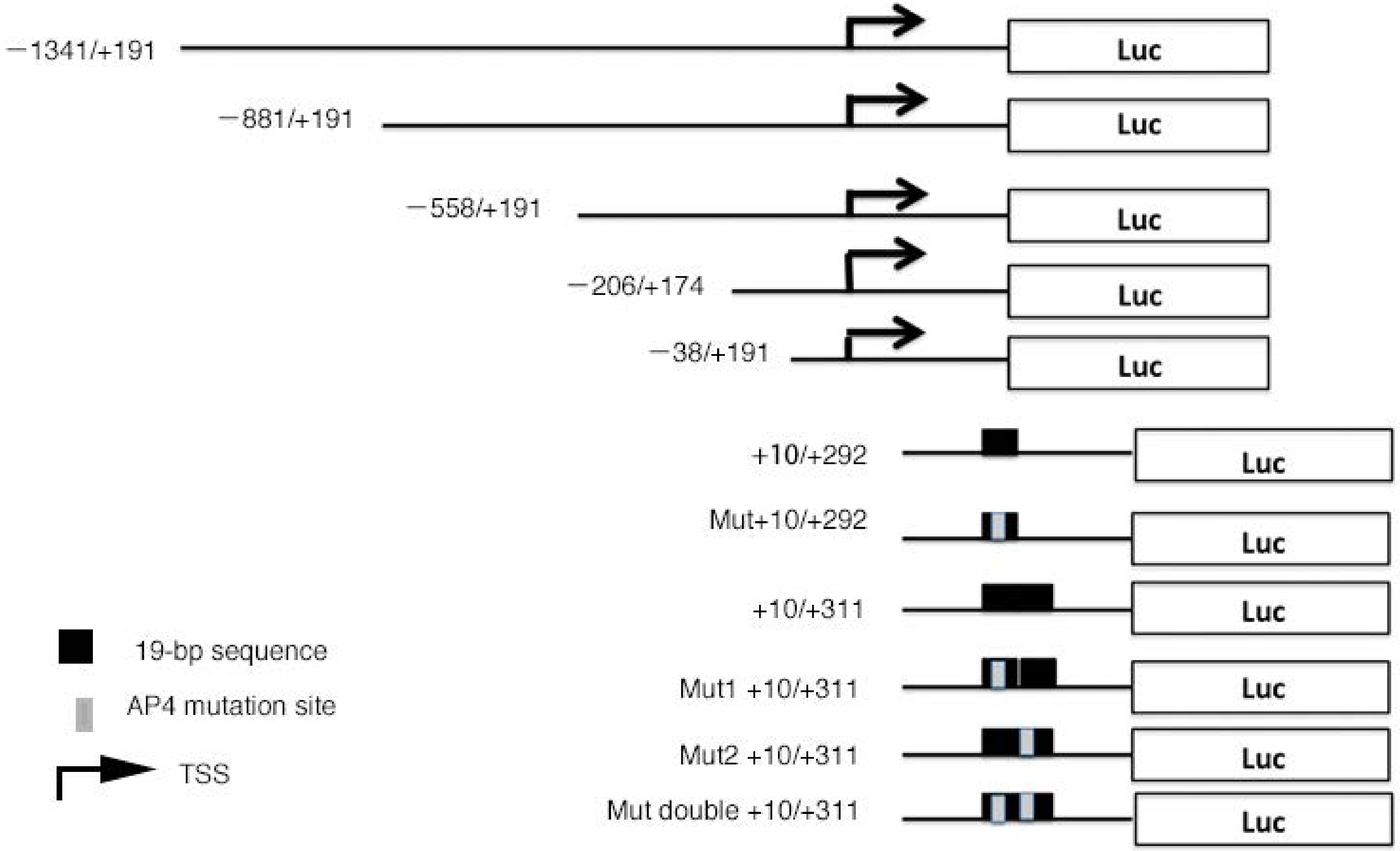
−1341/+191
Luc
−881/+191
Luc
−558/+191
Luc
−206/+174
Luc
−38/+191
Luc
+10/+292
Luc
Mut+10/+292
Luc
+10/+311
Luc
Mut1 +10/+311
Luc
Mut2 +10/+311
Luc
Mut double +10/+311
Luc
19-bp sequence
AP4 mutation site
TSS

FigureS9. Eleven kinds of plasmids transfected into cells.
