# Supplementary material for: AP4 positively regulates LAPTM4B to promote hepatocellular carcinoma growth and metastasis, while reducing chemotherapy sensitivity
Source: Mol Oncol. 2018 Feb 6;12(3):373–90. doi: 10.1002/1878-0261.12171 (PMC5830630; doi:10.1002/1878-0261.12171)
Supplement: Supplementary file 2 — Table S1. LAPTM4B*1 allele transcription factor prediction results of online database. Table S2. LAPTM4B*2 allele transcription factor prediction results of online database. Table S3. Primers for luciferase plasmids construction. Table S4. The siRNA target sequences of AP4. Table S5. The primer of AP4, LAPTM4B and GAPDH. Table S6. Antibodies used in WB. Table S7. Relationship between AP4 expression and clinicopathological features of HCC. Table S8. Relationship between LAPTM4B‐35 expression and clinicopathological features of HCC. [file MOL2-12-373-s002.pdf]

Table S1. LAPTM4B*1 allele transcription factor prediction results of online database

Sequence name	Factor name	Start position	End position	Dissimilarity	String	RE equally	RE query
LAPTM4B*1 allele	VDR [T00885]	1	4	0	GGGT	1.95703	2.66244
LAPTM4B*1 allele	VDR [T00885]	64	67	0	ACCC	1.95703	2.66244
LAPTM4B*1 allele	VDR [T00885]	208	211	0	ACCC	1.95703	2.66244
LAPTM4B*1 allele	VDR [T00885]	292	295	0	GGGT	1.95703	2.66244
LAPTM4B*1 allele	VDR [T00885]	319	322	0	GGGT	1.95703	2.66244
LAPTM4B*1 allele	VDR [T00885]	372	375	0	GGGT	1.95703	2.66244
LAPTM4B*1 allele	VDR [T00885]	442	445	0	GGGT	1.95703	2.66244
LAPTM4B*1 allele	VDR [T00885]	450	453	0	ACCC	1.95703	2.66244
LAPTM4B*1 allele	VDR [T00885]	475	478	0	GGGT	1.95703	2.66244
LAPTM4B*1 allele	WT1 I [T01840]	3	9	5.136498	GTGTGGT	0.18347	0.26637
LAPTM4B*1 allele	WT1 I [T01840]	85	91	8.128015	GTGAGCC	0.18347	0.27552
LAPTM4B*1 allele	WT1 I [T01840]	311	317	7.513446	TACTCAC	0.09174	0.09901
LAPTM4B*1 allele	ENKTF-1 [T00255]	9	16	13.885529	TGGCAAGC	0.27521	0.38844
LAPTM4B*1 allele	ENKTF-1 [T00255]	96	103	6.942764	TCGCGCCA	0.36694	0.56658
LAPTM4B*1 allele	ENKTF-1 [T00255]	229	236	12.629773	AACGGCCA	0.55042	0.66373
LAPTM4B*1 allele	ENKTF-1 [T00255]	285	292	1.255756	TGGCGGAG	0.06116	0.12299
LAPTM4B*1 allele	ENKTF-1 [T00255]	341	348	13.885529	TGGCTGGA	0.27521	0.38844
LAPTM4B*1 allele	ENKTF-1 [T00255]	378	385	6.942764	CATGGCCA	0.36694	0.56658
LAPTM4B*1 allele	ENKTF-1 [T00255]	380	387	6.942764	TGGCCAAG	0.36694	0.56658
LAPTM4B*1 allele	p300 [T01427]	23	27	1.540766	AATCC	0.48926	0.31279
LAPTM4B*1 allele	p300 [T01427]	50	54	3.482089	GGAGA	0.97852	1.62404
LAPTM4B*1 allele	p300 [T01427]	69	73	3.482089	GGAGG	0.97852	1.62404
LAPTM4B*1 allele	p300 [T01427]	75	79	3.482089	GGAGG	0.97852	1.62404
LAPTM4B*1 allele	p300 [T01427]	108	112	0.770383	ACTCC	0.48926	0.52181
LAPTM4B*1 allele	p300 [T01427]	170	174	5.408046	GGAAG	0.97852	1.11214
LAPTM4B*1 allele	p300 [T01427]	185	189	5.795432	GGAAC	0.48926	0.59067
LAPTM4B*1 allele	p300 [T01427]	218	222	0.770383	GGAGT	0.48926	0.52181
LAPTM4B*1 allele	p300 [T01427]	247	251	5.408046	GGAAG	0.97852	1.11214
LAPTM4B*1 allele	p300 [T01427]	256	260	3.099092	GGACC	0.48926	0.8339
LAPTM4B*1 allele	p300 [T01427]	259	263	3.482089	CCTCC	0.97852	1.62404
LAPTM4B*1 allele	p300 [T01427]	281	285	0	GGACT	0.48926	0.50476
LAPTM4B*1 allele	p300 [T01427]	289	293	3.482089	GGAGG	0.97852	1.62404
LAPTM4B*1 allele	p300 [T01427]	306	310	1.540766	GGATT	0.48926	0.31279
LAPTM4B*1 allele	p300 [T01427]	327	331	3.869475	GGAGC	0.48926	0.88508
LAPTM4B*1 allele	p300 [T01427]	330	334	3.869475	GCTCC	0.48926	0.88508
LAPTM4B*1 allele	p300 [T01427]	346	350	3.869475	GGAGC	0.48926	0.88508
LAPTM4B*1 allele	p300 [T01427]	366	370	0	GGACT	0.48926	0.50476
LAPTM4B*1 allele	p300 [T01427]	386	390	0	AGTCC	0.48926	0.50476
LAPTM4B*1 allele	p300 [T01427]	400	404	3.482089	CCTCC	0.97852	1.62404
LAPTM4B*1 allele	p300 [T01427]	406	410	2.711705	CGTCC	1.46777	1.81715
LAPTM4B*1 allele	p300 [T01427]	434	438	3.869475	GGAGC	0.48926	0.88508
LAPTM4B*1 allele	p300 [T01427]	465	469	3.869475	GGAGC	0.48926	0.88508
LAPTM4B*1 allele	p300 [T01427]	484	488	3.482089	GGAGG	0.97852	1.62404
LAPTM4B*1 allele	R2 [T00712]	25	30	14.26934	TCCCAG	1.46777	1.68655
LAPTM4B*1 allele	R2 [T00712]	47	52	11.337888	GCAGGA	0.61157	0.85803
LAPTM4B*1 allele	R2 [T00712]	72	77	9.512894	GGCGGA	1.83472	2.05292
LAPTM4B*1 allele	R2 [T00712]	110	115	1.824994	TCCAGC	0.12231	0.15544
LAPTM4B*1 allele	R2 [T00712]	167	172	14.26934	AAGGGA	1.46777	1.68655
LAPTM4B*1 allele	R2 [T00712]	215	220	9.512894	AATGGA	1.83472	2.05292
LAPTM4B*1 allele	R2 [T00712]	244	249	9.512894	GAAGGA	1.83472	2.05292
LAPTM4B*1 allele	R2 [T00712]	261	266	9.512894	TCCACT	1.83472	2.05292
LAPTM4B*1 allele	R2 [T00712]	278	283	14.26934	AGGGGA	1.46777	1.68655
LAPTM4B*1 allele	R2 [T00712]	286	291	9.512894	GGCGGA	1.83472	2.05292
LAPTM4B*1 allele	R2 [T00712]	303	308	0	GATGGA	0.36694	0.36637
LAPTM4B*1 allele	R2 [T00712]	324	329	9.512894	CTTGGA	1.83472	2.05292
LAPTM4B*1 allele	R2 [T00712]	332	337	1.824994	TCCAGC	0.12231	0.15544
LAPTM4B*1 allele	R2 [T00712]	343	348	1.824994	GCTGGA	0.12231	0.15544
LAPTM4B*1 allele	R2 [T00712]	388	393	9.512894	TCCGCC	1.83472	2.05292
LAPTM4B*1 allele	R2 [T00712]	402	407	14.26934	TCCCCG	1.46777	1.68655
LAPTM4B*1 allele	R2 [T00712]	408	413	14.26934	TCCCCG	1.46777	1.68655
LAPTM4B*1 allele	R2 [T00712]	431	436	14.26934	TTCGGA	1.46777	1.68655
LAPTM4B*1 allele	Ik-1 [T02702]	25	37	4.748597	TCCCAGCTACTCG	0.00078	0.00152
LAPTM4B*1 allele	AIRE [T05990]	39	48	10.110876	TTGGTTGGGC	0.04109	0.02997
LAPTM4B*1 allele	Elk-1 [T00250]	23	27	6.853012	AATCC	1.46777	1.47544
LAPTM4B*1 allele	Elk-1 [T00250]	50	54	7.213232	GGAGA	1.46777	1.47544
LAPTM4B*1 allele	Elk-1 [T00250]	69	73	5.092035	GGAGG	0.48926	0.96615
LAPTM4B*1 allele	Elk-1 [T00250]	75	79	5.092035	GGAGG	0.48926	0.96615
LAPTM4B*1 allele	Elk-1 [T00250]	108	112	7.984576	ACTCC	0.48926	0.52181
LAPTM4B*1 allele	Elk-1 [T00250]	170	174	0	GGAAG	0.48926	0.65789
LAPTM4B*1 allele	Elk-1 [T00250]	185	189	2.314032	GGAAC	0.48926	0.59067
LAPTM4B*1 allele	Elk-1 [T00250]	218	222	7.984576	GGAGT	0.48926	0.52181
LAPTM4B*1 allele	Elk-1 [T00250]	247	251	0	GGAAG	0.48926	0.65789
LAPTM4B*1 allele	Elk-1 [T00250]	256	260	6.274504	GGACC	0.97852	1.33867
LAPTM4B*1 allele	Elk-1 [T00250]	259	263	5.092035	CCTCC	0.48926	0.96615
LAPTM4B*1 allele	Elk-1 [T00250]	281	285	6.853012	GGACT	1.46777	1.47544
LAPTM4B*1 allele	Elk-1 [T00250]	289	293	5.092035	GGAGG	0.48926	0.96615
LAPTM4B*1 allele	Elk-1 [T00250]	306	310	6.853012	GGATT	1.46777	1.47544
LAPTM4B*1 allele	Elk-1 [T00250]	327	331	7.406067	GGAGC	0.48926	0.88508
LAPTM4B*1 allele	Elk-1 [T00250]	330	334	7.406067	GCTCC	0.48926	0.88508
LAPTM4B*1 allele	Elk-1 [T00250]	346	350	7.406067	GGAGC	0.48926	0.88508
LAPTM4B*1 allele	Elk-1 [T00250]	366	370	6.853012	GGACT	1.46777	1.47544
LAPTM4B*1 allele	Elk-1 [T00250]	386	390	6.853012	AGTCC	1.46777	1.47544
LAPTM4B*1 allele	Elk-1 [T00250]	400	404	5.092035	CCTCC	0.48926	0.96615
LAPTM4B*1 allele	Elk-1 [T00250]	406	410	3.960472	CGTCC	0.97852	1.40689
LAPTM4B*1 allele	Elk-1 [T00250]	434	438	7.406067	GGAGC	0.48926	0.88508
LAPTM4B*1 allele	Elk-1 [T00250]	465	469	7.406067	GGAGC	0.48926	0.88508

LAPTM4B*1 allele	Elk-1 [T00250]	484	488	5.092035	GGAGG	0.48926	0.96615
LAPTM4B*1 allele	-beta:RXR-alpha [T0	56	67	12.463326	TCGCTTGAACCC	0.03279	0.06694
LAPTM4B*1 allele	Nkx2-1 [T00857]	67	73	1.361249	CGGGAGG	0.09174	0.23205
LAPTM4B*1 allele	Nkx2-1 [T00857]	73	79	4.033796	GCGGAGG	0.21405	0.33738
LAPTM4B*1 allele	Nkx2-1 [T00857]	259	265	2.672547	CCTCCAC	0.03058	0.05146
LAPTM4B*1 allele	Nkx2-1 [T00857]	287	293	4.033796	GCGGAGG	0.21405	0.33738
LAPTM4B*1 allele	Nkx2-1 [T00857]	400	406	4.033796	CCTCCCC	0.21405	0.33738
LAPTM4B*1 allele	Nkx2-1 [T00857]	479	485	4.912497	ATCGAGG	0.4281	0.44062
LAPTM4B*1 allele	Nkx2-1 [T00857]	482	488	4.033796	GAGGAGG	0.21405	0.33738
LAPTM4B*1 allele	AP-2alphaA [T00035	17	22	1.437215	GCCTGT	0.12231	0.17171
LAPTM4B*1 allele	AP-2alphaA [T00035	69	74	2.003202	GGAGGC	0.24463	0.54059
LAPTM4B*1 allele	AP-2alphaA [T00035	114	119	0.159691	GCCTGG	0.24463	0.50933
LAPTM4B*1 allele	AP-2alphaA [T00035	248	253	3.004803	GAAGGC	0.36694	0.51621
LAPTM4B*1 allele	AP-2alphaA [T00035	428	433	3.004803	GCCTTC	0.36694	0.51621
LAPTM4B*1 allele	AP-2alphaA [T00035	484	489	2.003202	GGAGGC	0.24463	0.54059
LAPTM4B*1 allele	AP-2alphaA [T00035	488	493	0	GCAGGC	0.24463	0.50933
LAPTM4B*1 allele	Sp1 [T00759]	69	77	9.532578	GGAGGCGGA	0.0516	0.15696
LAPTM4B*1 allele	Sp1 [T00759]	283	291	11.706922	ACTGGCGGA	0.04205	0.08604
LAPTM4B*1 allele	Sp1 [T00759]	388	396	2.421386	TCCGCCCCG	0.02102	0.08184
LAPTM4B*1 allele	Sp1 [T00759]	393	401	0	CCCGCCCCC	0.00764	0.04235
LAPTM4B*1 allele	Sp1 [T00759]	410	418	8.565378	CCCGCCGCT	0.0344	0.11197
LAPTM4B*1 allele	C/EBPalpha [T00108	9	14	3.200469	TGGCAA	0.73389	0.79063
LAPTM4B*1 allele	C/EBPalpha [T00108	80	85	0.527529	TTGCAG	0.24463	0.15503
LAPTM4B*1 allele	C/EBPalpha [T00108	104	109	0	TTGCAC	0.24463	0.16663
LAPTM4B*1 allele	C/EBPalpha [T00108	179	184	3.022085	TCGCAA	0.73389	0.79063
LAPTM4B*1 allele	C/EBPbeta [T00581]	8	14	4.807336	GTGGCAA	0.24463	0.20486
LAPTM4B*1 allele	C/EBPbeta [T00581]	80	86	4.329855	TTGCAGT	0.21405	0.15729
LAPTM4B*1 allele	C/EBPbeta [T00581]	104	110	0.805985	TTGCACT	0.09174	0.07372
LAPTM4B*1 allele	C/EBPbeta [T00581]	178	184	4.807336	ATCGCAA	0.24463	0.20486
LAPTM4B*1 allele	E2F-1:DP-1 [T05204	95	103	2.513294	ATCGCGCCA	0.00573	0.01062
LAPTM4B*1 allele	E2F-1:DP-1 [T05204	285	293	14.802692	TGGCGGAGG	0.03058	0.07522
LAPTM4B*1 allele	E2F-1:DP-1 [T05204	391	399	12.662304	GCCCCGCCC	0.07262	0.20589
LAPTM4B*1 allele	E2F-1:DP-1 [T05204	408	416	10.023625	TCCCCGCCG	0.04396	0.11207
LAPTM4B*1 allele	CUTL1 [T00100]	103	108	2.06853	ATTGCA	0.73389	0.4205
LAPTM4B*1 allele	BTEB3 [T05051]	71	79	3.496398	AGGCGGAGG	0.01911	0.03439
LAPTM4B*1 allele	BTEB3 [T05051]	108	116	14.933149	ACTCCAGCC	0.06116	0.09755
LAPTM4B*1 allele	BTEB3 [T05051]	214	222	12.17827	GAATGGAGT	0.08791	0.13551
LAPTM4B*1 allele	BTEB3 [T05051]	259	267	7.847399	CCTCCACTC	0.05733	0.11349
LAPTM4B*1 allele	BTEB3 [T05051]	285	293	11.761034	TGGCGGAGG	0.08791	0.13551
LAPTM4B*1 allele	BTEB3 [T05051]	400	408	5.813911	CCTCCCCGT	0.01147	0.01611
LAPTM4B*1 allele	E47 [T00207]	26	32	4.682492	CCCAGCT	0.24463	0.35632
LAPTM4B*1 allele	E47 [T00207]	110	116	4.327662	TCCAGCC	0.24463	0.35632
LAPTM4B*1 allele	E47 [T00207]	114	120	5.305872	GCCTGGG	0.21405	0.30154
LAPTM4B*1 allele	E47 [T00207]	233	239	0	GCCAGAT	0.06116	0.08
LAPTM4B*1 allele	E47 [T00207]	282	288	4.485315	GACTGGC	0.24463	0.35632
LAPTM4B*1 allele	E47 [T00207]	332	338	5.510905	TCCAGCA	0.21405	0.30154
LAPTM4B*1 allele	E47 [T00207]	338	344	3.634763	AGCTGGC	0.03058	0.04668
LAPTM4B*1 allele	E47 [T00207]	342	348	4.327662	GGCTGGA	0.24463	0.35632
LAPTM4B*1 allele	SRY [T00997]	121	127	6.723683	CGACAAG	0.24463	0.2844
LAPTM4B*1 allele	MBF1 [T00492]	141	151	14.636473	CTCAAAAAAAA	0.05805	0.0185
LAPTM4B*1 allele	MBF1 [T00492]	192	202	14.636473	GACTAAAAGAA	0.05805	0.0185
LAPTM4B*1 allele	STAT5A [T04683]	170	173	0	GGAA	1.95703	2.04421
LAPTM4B*1 allele	STAT5A [T04683]	185	188	0	GGAA	1.95703	2.04421
LAPTM4B*1 allele	STAT5A [T04683]	247	250	0	GGAA	1.95703	2.04421
LAPTM4B*1 allele	POU3F2 [T00630]	176	182	9.725533	AAATCGC	0.06116	0.04046
LAPTM4B*1 allele	POU3F2 [T00630]	305	311	0.252682	TGGATTT	0.18347	0.05356
LAPTM4B*1 allele	FOXP3 [T04280]	175	183	14.668224	AAAATCGCA	0.00764	0.00401
LAPTM4B*1 allele	PEA3 [T00684]	182	189	8.315494	CAAGGAAC	0.09174	0.0533
LAPTM4B*1 allele	PEA3 [T00684]	244	251	4.375434	GAAGGAAG	0.01529	0.01882
LAPTM4B*1 allele	c-Ets-2 [T00113]	184	191	2.827022	AGGAACTA	0.09174	0.07753
LAPTM4B*1 allele	c-Ets-2 [T00113]	246	253	4.629332	AGGAAGGC	0.1376	0.14255
LAPTM4B*1 allele	c-Myb [T00137]	186	193	13.847077	GAACTAGA	0.06116	0.03125
LAPTM4B*1 allele	c-Myb [T00137]	217	224	12.979731	TGGAGTTA	0.1376	0.0721
LAPTM4B*1 allele	AP-3 (2) [T00039]	194	200	11.290546	CTAAAAG	0.36694	0.19834
LAPTM4B*1 allele	HNF-3beta [T02513]	195	201	3.504129	TAAAAGA	0.30579	0.1321
LAPTM4B*1 allele	HNF-3beta [T02513]	306	312	3.66377	GGATTTA	0.30579	0.1321
LAPTM4B*1 allele	YY1 [T00915]	101	107	7.55849	CCATTGC	0.33636	0.33922
LAPTM4B*1 allele	YY1 [T00915]	213	219	8.35288	TGAATGG	0.30579	0.25237
LAPTM4B*1 allele	YY1 [T00915]	301	307	7.441476	CTGATGG	0.33636	0.33922
LAPTM4B*1 allele	YY1 [T00915]	376	382	3.973697	CACATGG	0.12231	0.09733
LAPTM4B*1 allele	Hlf [T01071]	221	229	6.667463	GTTACACGA	0.02485	0.01102
LAPTM4B*1 allele	Myf-3 [T00519]	234	241	2.45354	CCAGATGA	0.10703	0.07462
LAPTM4B*1 allele	Myf-3 [T00519]	336	343	0	GCAGCTGG	0.1376	0.19853
LAPTM4B*1 allele	MyoD [T00525]	234	241	2.45354	CCAGATGA	0.10703	0.07462
LAPTM4B*1 allele	MyoD [T00525]	336	343	0	GCAGCTGG	0.1376	0.19853
LAPTM4B*1 allele	NF-X3 [T01514]	235	242	5.226274	CAGATGAA	0.08409	0.06977
LAPTM4B*1 allele	NF-X3 [T01514]	456	463	8.793097	CAGAAGCT	0.1376	0.13215
LAPTM4B*1 allele	LCR-F1 [T01599]	238	243	5.346725	ATGAAA	0.61157	0.32824
LAPTM4B*1 allele	LCR-F1 [T01599]	356	361	0.029165	ATGACG	0.48926	0.32166
LAPTM4B*1 allele	p53 [T00671]	45	51	0.525867	GGGCAGG	0.06116	0.15019
LAPTM4B*1 allele	p53 [T00671]	118	124	8.914655	GGGCGAC	0.30579	0.66358
LAPTM4B*1 allele	p53 [T00671]	249	255	9.164941	AAGGCCC	0.30579	0.66358
LAPTM4B*1 allele	p53 [T00671]	269	275	7.959461	GGGCCGA	0.18347	0.35414
LAPTM4B*1 allele	p53 [T00671]	346	352	9.966389	GGAGCCC	0.12231	0.36584
LAPTM4B*1 allele	p53 [T00671]	388	394	7.700559	TCCGCCC	0.12231	0.23097
LAPTM4B*1 allele	p53 [T00671]	393	399	3.891597	CCCGCCC	0.18347	0.50311
LAPTM4B*1 allele	p53 [T00671]	489	495	3.624632	CAGGCCC	0.09174	0.19797
LAPTM4B*1 allele	EIIaE-A [T00246]	43	49	14.472869	TTGGGCA	0.79504	1.44055
LAPTM4B*1 allele	EIIaE-A [T00246]	66	72	10.118218	CCGGGAG	0.55042	1.06004

LAPTM4B*1 allele	EIIaE-A [T00246]	116	122	10.118218	CTGGGCG	0.55042	1.06004
LAPTM4B*1 allele	EIIaE-A [T00246]	167	173	8.709302	AAGGGAA	0.55042	1.18679
LAPTM4B*1 allele	EIIaE-A [T00246]	251	257	14.472869	GGCCCGG	0.79504	1.44055
LAPTM4B*1 allele	EIIaE-A [T00246]	267	273	10.118218	CAGGGCC	0.55042	1.06004
LAPTM4B*1 allele	EIIaE-A [T00246]	277	283	14.472869	TAGGGGA	0.79504	1.44055
LAPTM4B*1 allele	EIIaE-A [T00246]	290	296	8.709302	GAGGGTG	0.55042	1.18679
LAPTM4B*1 allele	EIIaE-A [T00246]	317	323	14.472869	CCGGGTG	0.79504	1.44055
LAPTM4B*1 allele	EIIaE-A [T00246]	348	354	13.063953	AGCCCGC	0.79504	1.59022
LAPTM4B*1 allele	EIIaE-A [T00246]	390	396	10.118218	CGCCCCG	0.55042	1.06004
LAPTM4B*1 allele	EIIaE-A [T00246]	395	401	8.709302	CGCCCCC	0.55042	1.18679
LAPTM4B*1 allele	EIIaE-A [T00246]	397	403	8.709302	CCCCCTC	0.55042	1.18679
LAPTM4B*1 allele	EIIaE-A [T00246]	401	407	14.472869	CTCCCCG	0.79504	1.44055
LAPTM4B*1 allele	EIIaE-A [T00246]	440	446	13.063953	AAGGGTA	0.79504	1.59022
LAPTM4B*1 allele	EIIaE-A [T00246]	491	497	13.063953	GGCCCGC	0.79504	1.59022
LAPTM4B*1 allele	NHP-1 [T00621]	256	262	9.83	GGACCTC	0.45868	0.37409
LAPTM4B*1 allele	NHP-1 [T00621]	357	363	9.83	TGACGTC	0.45868	0.37409
LAPTM4B*1 allele	NHP-1 [T00621]	358	364	9.83	GACGTCA	0.45868	0.37409
LAPTM4B*1 allele	NHP-1 [T00621]	371	377	6.571346	CGGGTCA	0.09174	0.12311
LAPTM4B*1 allele	TFIIB [T00818]	263	270	11.657154	CACTCAGG	0.19876	0.28089
LAPTM4B*1 allele	XPF-1 [T00906]	263	271	10.392427	CACTCAGGG	0.1376	0.20921
LAPTM4B*1 allele	EBF [T05427]	263	273	6.8994	CACTCAGGGCC	0.00764	0.01846
LAPTM4B*1 allele	MZF-1 [T00529]	277	283	2.220412	TAGGGGA	0.12231	0.16327
LAPTM4B*1 allele	MZF-1 [T00529]	402	408	1.166538	TCCCCGT	0.12231	0.21277
LAPTM4B*1 allele	MZF-1 [T00529]	408	414	0.112664	TCCCCGC	0.12231	0.3098
LAPTM4B*1 allele	NF-kappaB1 [T00593	279	290	14.056581	GGGGACTGGCGG	0.01224	0.02439
LAPTM4B*1 allele	Pax-5 [T00070]	295	301	7.762216	TGCACGC	0.06116	0.12167
LAPTM4B*1 allele	AhR:Arnt [T05394]	297	305	9.325207	CACGCTGAT	0.0516	0.05428
LAPTM4B*1 allele	U2F2 (Oct-2.1) [T006	172	179	8.36845	AAGAAAAT	0.06116	0.01676
LAPTM4B*1 allele	U2F2 (Oct-2.1) [T006	308	315	11.697988	ATTTACTC	0.05351	0.01894
LAPTM4B*1 allele	RAR-gamma [T00720	311	318	5.085116	TACTCACC	0.06116	0.06447
LAPTM4B*1 allele	Myf-5 [T00521]	329	341	11.871492	AGCTCCAGCAGCT	0.01615	0.02459
LAPTM4B*1 allele	Myf-5 [T00521]	332	344	7.122895	TCCAGCAGCTGGC	0.00267	0.00396
LAPTM4B*1 allele	Myf-5 [T00521]	415	427	14.24579	CGCTGCAGCGGTC	0.03006	0.04866
LAPTM4B*1 allele	AP-4 [T00036]	333	343	1.268101	CCAGCAGCTGG	0.0037	0.00546
LAPTM4B*1 allele	Tal-1 [T00790]	332	342	14.153038	TCCAGCAGCTG	0.0387	0.04451
LAPTM4B*1 allele	Tal-1 [T00790]	337	347	8.412013	CAGCTGGCTGG	0.01935	0.02734
LAPTM4B*1 allele	DP-1 [T01548]	346	355	14.492168	GGAGCCCGCG	0.0516	0.16567
LAPTM4B*1 allele	DP-1 [T01548]	406	415	12.705831	CGTCCCCGCC	0.0516	0.14405
LAPTM4B*1 allele	E2F-1 [T01542]	72	78	5.331496	GGCGGAG	0.12231	0.41733
LAPTM4B*1 allele	E2F-1 [T01542]	286	292	5.331496	GGCGGAG	0.12231	0.41733
LAPTM4B*1 allele	E2F-1 [T01542]	349	355	5.944817	GCCCGCG	0.12231	0.39652
LAPTM4B*1 allele	E2F-1 [T01542]	387	393	7.879274	GTCCGCC	0.30579	0.74391
LAPTM4B*1 allele	E2F-1 [T01542]	392	398	3.397038	CCCCGCC	0.18347	0.43342
LAPTM4B*1 allele	E2F-1 [T01542]	409	415	3.397038	CCCCGCC	0.18347	0.43342
LAPTM4B*1 allele	E2F-1 [T01542]	412	418	7.060495	CGCCGCT	0.12231	0.26595
LAPTM4B*1 allele	E2F-1 [T01542]	421	427	10.575502	AGCGGTC	0.12231	0.15928
LAPTM4B*1 allele	E2F-1 [T01542]	492	498	5.944817	GCCCGCG	0.12231	0.39652
LAPTM4B*1 allele	E2F-1 [T01543]	350	358	4.354054	CCCGCGATG	0.0688	0.13723
LAPTM4B*1 allele	E2F-1 [T01543]	393	401	6.363515	CCCGCCCCC	0.0516	0.23697
LAPTM4B*1 allele	E2F-1 [T01543]	410	418	6.570994	CCCGCCGCT	0.0516	0.23697
LAPTM4B*1 allele	E2F-1 [T01543]	492	500	7.224594	GCCCGCGGG	0.0344	0.13242
LAPTM4B*1 allele	USF2b [T02377]	353	360	12.629773	GCGATGAC	0.55042	0.43861
LAPTM4B*1 allele	USF2b [T02377]	361	368	11.374018	GTCACGGA	0.27521	0.34414
LAPTM4B*1 allele	USF2b [T02377]	374	381	1.255756	GTCACATG	0.06116	0.04568
LAPTM4B*1 allele	c-Jun [T00132]	357	363	4.469954	TGACGTC	0.21405	0.18571
LAPTM4B*1 allele	c-Jun [T00132]	358	364	4.469954	GACGTCA	0.21405	0.18571
LAPTM4B*1 allele	c-Jun [T00132]	371	377	4.522753	CGGGTCA	0.21405	0.18571
LAPTM4B*1 allele	CREMalpha [T01803	353	364	7.13467	GCGATGACGTCA	0.00753	0.0043
LAPTM4B*1 allele	CREMalpha [T01803	357	368	5.668944	TGACGTCACGGA	0.00251	0.00183
LAPTM4B*1 allele	CREB [T00163]	356	364	0.032925	ATGACGTCA	0.00764	0.00545
LAPTM4B*1 allele	CREB [T00163]	357	365	0.024694	TGACGTCAC	0.00764	0.00545
LAPTM4B*1 allele	ATF-2 [T00167]	356	364	0.034254	ATGACGTCA	0.00764	0.00545
LAPTM4B*1 allele	ATF-2 [T00167]	357	365	0	TGACGTCAC	0.00764	0.00545
LAPTM4B*1 allele	ATF [T00051]	356	364	0.462475	ATGACGTCA	0.00956	0.00658
LAPTM4B*1 allele	ATF [T00051]	357	365	0.313871	TGACGTCAC	0.00956	0.00658
LAPTM4B*1 allele	E4F [T00222]	357	365	12.100443	TGACGTCAC	0.04587	0.02801
LAPTM4B*1 allele	c-Fos [T00123]	356	363	10.285697	ATGACGTC	0.12231	0.0851
LAPTM4B*1 allele	c-Fos [T00123]	358	365	11.816087	GACGTCAC	0.0688	0.04757
LAPTM4B*1 allele	c-Fos [T00123]	371	378	11.455199	CGGGTCAC	0.10703	0.10587
LAPTM4B*1 allele	ATF-1 [T00968]	355	365	2.828372	GATGACGTCAC	0.00167	0.00189
LAPTM4B*1 allele	ATF-1 [T00968]	356	366	3.669682	ATGACGTCACG	0.0043	0.00406
LAPTM4B*1 allele	T3R-alpha1 [T01152]	367	377	13.79108	GACTCGGGTCA	0.08314	0.06654
LAPTM4B*1 allele	ER-beta [T04651]	369	377	9.122536	CTCGGGTCA	0.00764	0.00888
LAPTM4B*1 allele	RAR-beta [T00721]	370	377	2.799654	TCGGGTCA	0.05351	0.08056
LAPTM4B*1 allele	ER-alpha [T00261]	371	378	1.367305	CGGGTCAC	0.09938	0.09901
LAPTM4B*1 allele	NFI/CTF [T00094]	35	42	2.683003	TCGGTTGG	0.01529	0.02455
LAPTM4B*1 allele	NFI/CTF [T00094]	39	46	8.241664	TTGGTTGG	0.04587	0.05391
LAPTM4B*1 allele	NFI/CTF [T00094]	321	328	5.558661	GTGCTTGG	0.1376	0.11996
LAPTM4B*1 allele	NFI/CTF [T00094]	383	390	13.146004	CCAAGTCC	0.27521	0.1551
LAPTM4B*1 allele	Sp3 [T02338]	387	395	5.19869	GTCCGCCCC	0.02293	0.08583
LAPTM4B*1 allele	Sp3 [T02338]	392	400	0	CCCCGCCCC	0.00191	0.01352
LAPTM4B*1 allele	GCF [T00320]	390	400	5.946992	CGCCCCGCCCC	0.00323	0.01919
LAPTM4B*1 allele	MAZ [T00490]	393	403	14.391959	CCCGCCCCCTC	0.03512	0.15075
LAPTM4B*1 allele	MAZ [T00490]	399	409	1.454467	CCCTCCCCGTC	0.00418	0.01681
LAPTM4B*1 allele	MAZ [T00490]	405	415	14.561456	CCGTCCCCGCC	0.03512	0.15075
LAPTM4B*1 allele	WT1 [T00899]	395	405	7.524526	CGCCCCCTCCC	0.00382	0.02276
LAPTM4B*1 allele	WT1 [T00899]	406	416	11.883285	CGTCCCCGCCG	0.0258	0.10484
LAPTM4B*1 allele	R1 [T00711]	287	298	11.545139	GCGGAGGGTGCA	0.0129	0.03427
LAPTM4B*1 allele	R1 [T00711]	395	406	8.256952	CGCCCCCTCCCC	0.00932	0.02482

LAPTM4B*1 allele	LF-A1 [T00467]	7	13	9.701266	GGTGGCA	0.55042	0.74465
LAPTM4B*1 allele	LF-A1 [T00467]	43	49	1.561557	TTGGGCA	0.27521	0.49037
LAPTM4B*1 allele	LF-A1 [T00467]	451	457	8.920488	CCCGGCA	0.36694	0.51402
LAPTM4B*1 allele	LF-A1 [T00467]	484	490	5.631412	GGAGGCA	0.27521	0.32654
LAPTM4B*1 allele	GATA-1 [T00306]	476	481	1.908093	GGTATC	0.73389	0.35601
LAPTM4B*1 allele	WT1 I -KTS [T00900	347	356	14.457704	GAGCCCGCGA	0.04491	0.19021
LAPTM4B*1 allele	WT1 I -KTS [T00900	394	403	10.053856	CCGCCCCCTC	0.00764	0.04259
LAPTM4B*1 allele	WT1 I -KTS [T00900	405	414	14.586256	CCGTCCCCGC	0.04491	0.19021
LAPTM4B*1 allele	WT1 -KTS [T01839]	347	356	14.457704	GAGCCCGCGA	0.04491	0.19021
LAPTM4B*1 allele	WT1 -KTS [T01839]	394	403	10.053856	CCGCCCCCTC	0.00764	0.04259
LAPTM4B*1 allele	WT1 -KTS [T01839]	405	414	14.586256	CCGTCCCCGC	0.04491	0.19021
LAPTM4B*1 allele	IA-1 [T05887]	394	406	6.673112	CCGCCCCCTCCCC	0.00097	0.0038
LAPTM4B*1 allele	ETF [T00270]	387	395	1.261043	GTCCGCCCC	0.02867	0.09432
LAPTM4B*1 allele	ETF [T00270]	392	400	0	CCCCGCCCC	0.00191	0.01352
LAPTM4B*1 allele	NF-1 [T00539]	382	386	0	GCCAA	0.48926	0.54339
LAPTM4B*1 allele	USF2 [T00878]	373	381	3.706299	GGTCACATG	0.01147	0.0105
LAPTM4B*1 allele	T3R-beta1 [T00851]	370	380	13.382055	TCGGGTCACAT	0.0602	0.05079
LAPTM4B*1 allele	ARP-1 [T00045]	369	377	3.301012	CTCGGGTCA	0.00764	0.00821
LAPTM4B*1 allele	E4F1 [T00223]	357	365	0.123447	TGACGTCAC	0.03058	0.02893
LAPTM4B*1 allele	E12 [T00204]	332	344	14.548481	TCCAGCAGCTGGC	0.00753	0.01182
LAPTM4B*1 allele	TFII-I [T00824]	303	313	6.490932	GATGGATTTAC	0.00764	0.00718
LAPTM4B*1 allele	Pbx1b [T02087]	236	245	14.774452	AGATGAAAGA	0.0516	0.03184
LAPTM4B*1 allele	Pbx1b [T02087]	299	308	8.781358	CGCTGATGGA	0.0258	0.01017
LAPTM4B*1 allele	Arnt [T01346]	297	305	10.586083	CACGCTGAT	0.02293	0.03292
LAPTM4B*1 allele	AhR [T01795]	294	301	2.665859	GTGCACGC	0.11467	0.18662
LAPTM4B*1 allele	RCS1 [T03538]	290	301	9.535158	GAGGGTGCACGC	0.00376	0.00745
LAPTM4B*1 allele	HMG I(Y) [T02368]	170	176	5.151271	GGAAGAA	0.15289	0.11288
LAPTM4B*1 allele	HMG I(Y) [T02368]	185	191	4.951498	GGAACTA	0.15289	0.11288
LAPTM4B*1 allele	HMG I(Y) [T02368]	247	253	7.014009	GGAAGGC	0.15289	0.27476
LAPTM4B*1 allele	STAT4 [T01577]	170	175	4.411765	GGAAGA	0.48926	0.63417
LAPTM4B*1 allele	STAT4 [T01577]	185	190	1.470588	GGAACT	0.48926	0.39374
LAPTM4B*1 allele	STAT4 [T01577]	247	252	5.882353	GGAAGG	0.12231	0.21277
LAPTM4B*1 allele	c-Ets-1 [T00112]	168	174	6.471844	AGGGAAG	0.12231	0.173
LAPTM4B*1 allele	c-Ets-1 [T00112]	183	189	4.46609	AAGGAAC	0.15289	0.14506
LAPTM4B*1 allele	c-Ets-1 [T00112]	245	251	0.484609	AAGGAAG	0.09174	0.10593
LAPTM4B*1 allele	ox binding factor [T0(	239	248	8.695301	TGAAAGAAGG	0.0559	0.04456
LAPTM4B*1 allele	HSF1 (long) [T01042	210	217	0	CCTTGAAT	0.02293	0.01458
LAPTM4B*1 allele	HSF1 (short) [T02104	210	217	0	CCTTGAAT	0.02293	0.01458
LAPTM4B*1 allele	HNF-1B [T01950]	22	29	14.384751	TAATCCCA	0.10703	0.04856
LAPTM4B*1 allele	NFdeltaE3A [T00976]	4	13	13.153012	TGTGGTGGCA	0.0172	0.01577
LAPTM4B*1 allele	AML1a [T02256]	0	9	7.146755	CGGGTGTGGT	0.01386	0.01471

Table S2. LAPTM4B*2 allele transcription factor prediction results of online database

Sequence name	Factor name	Start position	End position	Dissimilarity	String	RE equally	RE query
LAPTM4B*2 allele	VDR [T00885]	1	4	0	GGGT	2.03125	2.78039
LAPTM4B*2 allele	VDR [T00885]	64	67	0	ACCC	2.03125	2.78039
LAPTM4B*2 allele	VDR [T00885]	208	211	0	ACCC	2.03125	2.78039
LAPTM4B*2 allele	VDR [T00885]	292	295	0	GGGT	2.03125	2.78039
LAPTM4B*2 allele	VDR [T00885]	319	322	0	GGGT	2.03125	2.78039
LAPTM4B*2 allele	VDR [T00885]	391	394	0	GGGT	2.03125	2.78039
LAPTM4B*2 allele	VDR [T00885]	461	464	0	GGGT	2.03125	2.78039
LAPTM4B*2 allele	VDR [T00885]	469	472	0	ACCC	2.03125	2.78039
LAPTM4B*2 allele	VDR [T00885]	494	497	0	GGGT	2.03125	2.78039
LAPTM4B*2 allele	WT1 I [T01840]	3	9	5.136498	GTGTGGT	0.19043	0.27782
LAPTM4B*2 allele	WT1 I [T01840]	85	91	8.128015	GTGAGCC	0.19043	0.2886
LAPTM4B*2 allele	WT1 I [T01840]	311	317	7.513446	TACTCAC	0.09521	0.10337
LAPTM4B*2 allele	ENKTF-1 [T00255]	9	16	13.885529	TGGCAAGC	0.28564	0.40596
LAPTM4B*2 allele	ENKTF-1 [T00255]	96	103	6.942764	TCGCGCCA	0.38086	0.5915
LAPTM4B*2 allele	ENKTF-1 [T00255]	229	236	12.629773	AACGGCCA	0.57129	0.68978
LAPTM4B*2 allele	ENKTF-1 [T00255]	285	292	1.255756	TGGCGGAG	0.06348	0.12883
LAPTM4B*2 allele	ENKTF-1 [T00255]	360	367	13.885529	TGGCTGGA	0.28564	0.40596
LAPTM4B*2 allele	ENKTF-1 [T00255]	397	404	6.942764	CATGGCCA	0.38086	0.5915
LAPTM4B*2 allele	ENKTF-1 [T00255]	399	406	6.942764	TGGCCAAG	0.38086	0.5915
LAPTM4B*2 allele	p300 [T01427]	23	27	1.540766	AATCC	0.50781	0.32684
LAPTM4B*2 allele	p300 [T01427]	50	54	3.482089	GGAGA	1.01562	1.66661
LAPTM4B*2 allele	p300 [T01427]	69	73	3.482089	GGAGG	1.01562	1.66661
LAPTM4B*2 allele	p300 [T01427]	75	79	3.482089	GGAGG	1.01562	1.66661
LAPTM4B*2 allele	p300 [T01427]	108	112	0.770383	ACTCC	0.50781	0.5441
LAPTM4B*2 allele	p300 [T01427]	170	174	5.408046	GGAAG	1.01562	1.12972
LAPTM4B*2 allele	p300 [T01427]	185	189	5.795432	GGAAC	0.50781	0.60764
LAPTM4B*2 allele	p300 [T01427]	218	222	0.770383	GGAGT	0.50781	0.5441
LAPTM4B*2 allele	p300 [T01427]	247	251	5.408046	GGAAG	1.01562	1.12972
LAPTM4B*2 allele	p300 [T01427]	256	260	3.099092	GGACC	0.50781	0.86923
LAPTM4B*2 allele	p300 [T01427]	259	263	3.482089	CCTCC	1.01562	1.66661
LAPTM4B*2 allele	p300 [T01427]	281	285	0	GGACT	0.50781	0.52761
LAPTM4B*2 allele	p300 [T01427]	289	293	3.482089	GGAGG	1.01562	1.66661
LAPTM4B*2 allele	p300 [T01427]	306	310	1.540766	GGATT	0.50781	0.32684
LAPTM4B*2 allele	p300 [T01427]	327	331	3.869475	GGAGC	0.50781	0.91795
LAPTM4B*2 allele	p300 [T01427]	330	334	3.869475	GCTCC	0.50781	0.91795
LAPTM4B*2 allele	p300 [T01427]	346	350	3.869475	GGAGC	0.50781	0.91795
LAPTM4B*2 allele	p300 [T01427]	349	353	3.869475	GCTCC	0.50781	0.91795
LAPTM4B*2 allele	p300 [T01427]	365	369	3.869475	GGAGC	0.50781	0.91795
LAPTM4B*2 allele	p300 [T01427]	385	389	0	GGACT	0.50781	0.52761
LAPTM4B*2 allele	p300 [T01427]	405	409	0	AGTCC	0.50781	0.52761
LAPTM4B*2 allele	p300 [T01427]	419	423	3.482089	CCTCC	1.01562	1.66661
LAPTM4B*2 allele	p300 [T01427]	425	429	2.711705	CGTCC	1.52344	1.87952
LAPTM4B*2 allele	p300 [T01427]	453	457	3.869475	GGAGC	0.50781	0.91795
LAPTM4B*2 allele	p300 [T01427]	484	488	3.869475	GGAGC	0.50781	0.91795
LAPTM4B*2 allele	p300 [T01427]	503	507	3.482089	GGAGG	1.01562	1.66661
LAPTM4B*2 allele	R2 [T00712]	25	30	14.26934	TCCCAG	1.52344	1.74102
LAPTM4B*2 allele	R2 [T00712]	47	52	11.337888	GCAGGA	0.63477	0.89231
LAPTM4B*2 allele	R2 [T00712]	72	77	9.512894	GGCGGA	1.9043	2.12254
LAPTM4B*2 allele	R2 [T00712]	110	115	1.824994	TCCAGC	0.12695	0.16258
LAPTM4B*2 allele	R2 [T00712]	167	172	14.26934	AAGGGA	1.52344	1.74102
LAPTM4B*2 allele	R2 [T00712]	215	220	9.512894	AATGGA	1.9043	2.12254
LAPTM4B*2 allele	R2 [T00712]	244	249	9.512894	GAAGGA	1.9043	2.12254
LAPTM4B*2 allele	R2 [T00712]	261	266	9.512894	TCCACT	1.9043	2.12254
LAPTM4B*2 allele	R2 [T00712]	278	283	14.26934	AGGGGA	1.52344	1.74102
LAPTM4B*2 allele	R2 [T00712]	286	291	9.512894	GGCGGA	1.9043	2.12254
LAPTM4B*2 allele	R2 [T00712]	303	308	0	GATGGA	0.38086	0.38152
LAPTM4B*2 allele	R2 [T00712]	324	329	9.512894	CTTGGA	1.9043	2.12254
LAPTM4B*2 allele	R2 [T00712]	332	337	1.824994	TCCAGC	0.12695	0.16258
LAPTM4B*2 allele	R2 [T00712]	343	348	9.512894	CTTGGA	1.9043	2.12254
LAPTM4B*2 allele	R2 [T00712]	351	356	1.824994	TCCAGC	0.12695	0.16258
LAPTM4B*2 allele	R2 [T00712]	362	367	1.824994	GCTGGA	0.12695	0.16258
LAPTM4B*2 allele	R2 [T00712]	407	412	9.512894	TCCGCC	1.9043	2.12254
LAPTM4B*2 allele	R2 [T00712]	421	426	14.26934	TCCCCG	1.52344	1.74102
LAPTM4B*2 allele	R2 [T00712]	427	432	14.26934	TCCCCG	1.52344	1.74102
LAPTM4B*2 allele	R2 [T00712]	450	455	14.26934	TTCGGA	1.52344	1.74102
LAPTM4B*2 allele	Ik-1 [T02702]	25	37	4.748597	TCCCAGCTACTCG	0.00081	0.00156
LAPTM4B*2 allele	AIRE [T05990]	39	48	10.110876	TTGGTTGGGC	0.04265	0.03058
LAPTM4B*2 allele	Elk-1 [T00250]	23	27	6.853012	AATCC	1.52344	1.52572

LAPTM4B*2 allele	Elk-1 [T00250]	50	54	7.213232	GGAGA	1.52344	1.52572
LAPTM4B*2 allele	Elk-1 [T00250]	69	73	5.092035	GGAGG	0.50781	0.99535
LAPTM4B*2 allele	Elk-1 [T00250]	75	79	5.092035	GGAGG	0.50781	0.99535
LAPTM4B*2 allele	Elk-1 [T00250]	108	112	7.984576	ACTCC	0.50781	0.5441
LAPTM4B*2 allele	Elk-1 [T00250]	170	174	0	GGAAG	0.50781	0.67126
LAPTM4B*2 allele	Elk-1 [T00250]	185	189	2.314032	GGAAC	0.50781	0.60764
LAPTM4B*2 allele	Elk-1 [T00250]	218	222	7.984576	GGAGT	0.50781	0.5441
LAPTM4B*2 allele	Elk-1 [T00250]	247	251	0	GGAAG	0.50781	0.67126
LAPTM4B*2 allele	Elk-1 [T00250]	256	260	6.274504	GGACC	1.01562	1.39684
LAPTM4B*2 allele	Elk-1 [T00250]	259	263	5.092035	CCTCC	0.50781	0.99535
LAPTM4B*2 allele	Elk-1 [T00250]	281	285	6.853012	GGACT	1.52344	1.52572
LAPTM4B*2 allele	Elk-1 [T00250]	289	293	5.092035	GGAGG	0.50781	0.99535
LAPTM4B*2 allele	Elk-1 [T00250]	306	310	6.853012	GGATT	1.52344	1.52572
LAPTM4B*2 allele	Elk-1 [T00250]	327	331	7.406067	GGAGC	0.50781	0.91795
LAPTM4B*2 allele	Elk-1 [T00250]	330	334	7.406067	GCTCC	0.50781	0.91795
LAPTM4B*2 allele	Elk-1 [T00250]	346	350	7.406067	GGAGC	0.50781	0.91795
LAPTM4B*2 allele	Elk-1 [T00250]	349	353	7.406067	GCTCC	0.50781	0.91795
LAPTM4B*2 allele	Elk-1 [T00250]	365	369	7.406067	GGAGC	0.50781	0.91795
LAPTM4B*2 allele	Elk-1 [T00250]	385	389	6.853012	GGACT	1.52344	1.52572
LAPTM4B*2 allele	Elk-1 [T00250]	405	409	6.853012	AGTCC	1.52344	1.52572
LAPTM4B*2 allele	Elk-1 [T00250]	419	423	5.092035	CCTCC	0.50781	0.99535
LAPTM4B*2 allele	Elk-1 [T00250]	425	429	3.960472	CGTCC	1.01562	1.46205
LAPTM4B*2 allele	Elk-1 [T00250]	453	457	7.406067	GGAGC	0.50781	0.91795
LAPTM4B*2 allele	Elk-1 [T00250]	484	488	7.406067	GGAGC	0.50781	0.91795
LAPTM4B*2 allele	Elk-1 [T00250]	503	507	5.092035	GGAGG	0.50781	0.99535
LAPTM4B*2 allele	R-beta:RXR-alpha [T05	56	67	12.463326	TCGCTTGAACCC	0.03403	0.0696
LAPTM4B*2 allele	Nkx2-1 [T00857]	67	73	1.361249	CGGGAGG	0.09521	0.23977
LAPTM4B*2 allele	Nkx2-1 [T00857]	73	79	4.033796	GCGGAGG	0.22217	0.34697
LAPTM4B*2 allele	Nkx2-1 [T00857]	259	265	2.672547	CCTCCAC	0.03174	0.05358
LAPTM4B*2 allele	Nkx2-1 [T00857]	287	293	4.033796	GCGGAGG	0.22217	0.34697
LAPTM4B*2 allele	Nkx2-1 [T00857]	419	425	4.033796	CCTCCCC	0.22217	0.34697
LAPTM4B*2 allele	Nkx2-1 [T00857]	498	504	4.912497	ATCGAGG	0.44434	0.45317
LAPTM4B*2 allele	Nkx2-1 [T00857]	501	507	4.033796	GAGGAGG	0.22217	0.34697
LAPTM4B*2 allele	AP-2alphaA [T00035]	17	22	1.437215	GCCTGT	0.12695	0.17759
LAPTM4B*2 allele	AP-2alphaA [T00035]	69	74	2.003202	GGAGGC	0.25391	0.56136
LAPTM4B*2 allele	AP-2alphaA [T00035]	114	119	0.159691	GCCTGG	0.25391	0.53157
LAPTM4B*2 allele	AP-2alphaA [T00035]	248	253	3.004803	GAAGGC	0.38086	0.53417
LAPTM4B*2 allele	AP-2alphaA [T00035]	447	452	3.004803	GCCTTC	0.38086	0.53417
LAPTM4B*2 allele	AP-2alphaA [T00035]	503	508	2.003202	GGAGGC	0.25391	0.56136
LAPTM4B*2 allele	AP-2alphaA [T00035]	507	512	0	GCAGGC	0.25391	0.53157
LAPTM4B*2 allele	Sp1 [T00759]	69	77	9.532578	GGAGGCGGA	0.05356	0.16378
LAPTM4B*2 allele	Sp1 [T00759]	283	291	11.706922	ACTGGCGGA	0.04364	0.08976
LAPTM4B*2 allele	Sp1 [T00759]	407	415	2.421386	TCCGCCCCG	0.02182	0.08543
LAPTM4B*2 allele	Sp1 [T00759]	412	420	0	CCCGCCCCC	0.00793	0.04405
LAPTM4B*2 allele	Sp1 [T00759]	429	437	8.565378	CCCGCCGCT	0.03571	0.1167
LAPTM4B*2 allele	C/EBPalpha [T00108]	9	14	3.200469	TGGCAA	0.76172	0.81548
LAPTM4B*2 allele	C/EBPalpha [T00108]	80	85	0.527529	TTGCAG	0.25391	0.16214
LAPTM4B*2 allele	C/EBPalpha [T00108]	104	109	0	TTGCAC	0.25391	0.17301
LAPTM4B*2 allele	C/EBPalpha [T00108]	179	184	3.022085	TCGCAA	0.76172	0.81548
LAPTM4B*2 allele	C/EBPbeta [T00581]	8	14	4.807336	GTGGCAA	0.25391	0.21138
LAPTM4B*2 allele	C/EBPbeta [T00581]	80	86	4.329855	TTGCAGT	0.22217	0.16143
LAPTM4B*2 allele	C/EBPbeta [T00581]	104	110	0.805985	TTGCACT	0.09521	0.0763
LAPTM4B*2 allele	C/EBPbeta [T00581]	178	184	4.807336	ATCGCAA	0.25391	0.21138
LAPTM4B*2 allele	E2F-1:DP-1 [T05204]	95	103	2.513294	ATCGCGCCA	0.00595	0.01109
LAPTM4B*2 allele	E2F-1:DP-1 [T05204]	285	293	14.802692	TGGCGGAGG	0.03174	0.07884
LAPTM4B*2 allele	E2F-1:DP-1 [T05204]	410	418	12.662304	GCCCCGCCC	0.07538	0.2151
LAPTM4B*2 allele	E2F-1:DP-1 [T05204]	427	435	10.023625	TCCCCGCCG	0.04562	0.11667
LAPTM4B*2 allele	CUTL1 [T00100]	103	108	2.06853	ATTGCA	0.76172	0.43815
LAPTM4B*2 allele	SXR:RXR-alpha [T0567	103	111	11.263007	ATTGCACTC	0.03174	0.02398
LAPTM4B*2 allele	BTEB3 [T05051]	71	79	3.496398	AGGCGGAGG	0.01984	0.03464
LAPTM4B*2 allele	BTEB3 [T05051]	108	116	14.933149	ACTCCAGCC	0.06348	0.10054
LAPTM4B*2 allele	BTEB3 [T05051]	214	222	12.17827	GAATGGAGT	0.09125	0.13877
LAPTM4B*2 allele	BTEB3 [T05051]	259	267	7.847399	CCTCCACTC	0.05951	0.11517
LAPTM4B*2 allele	BTEB3 [T05051]	285	293	11.761034	TGGCGGAGG	0.09125	0.13877
LAPTM4B*2 allele	BTEB3 [T05051]	419	427	5.813911	CCTCCCCGT	0.0119	0.01657
LAPTM4B*2 allele	E47 [T00207]	26	32	4.682492	CCCAGCT	0.25391	0.37276
LAPTM4B*2 allele	E47 [T00207]	110	116	4.327662	TCCAGCC	0.25391	0.37276
LAPTM4B*2 allele	E47 [T00207]	114	120	5.305872	GCCTGGG	0.22217	0.31418
LAPTM4B*2 allele	E47 [T00207]	233	239	0	GCCAGAT	0.06348	0.08332
LAPTM4B*2 allele	E47 [T00207]	282	288	4.485315	GACTGGC	0.25391	0.37276

LAPTM4B*2 allele	E47 [T00207]	332	338	5.510905	TCCAGCA	0.22217	0.31418
LAPTM4B*2 allele	E47 [T00207]	351	357	5.510905	TCCAGCA	0.22217	0.31418
LAPTM4B*2 allele	E47 [T00207]	357	363	3.634763	AGCTGGC	0.03174	0.04895
LAPTM4B*2 allele	E47 [T00207]	361	367	4.327662	GGCTGGA	0.25391	0.37276
LAPTM4B*2 allele	SRY [T00997]	121	127	6.723683	CGACAAG	0.25391	0.2916
LAPTM4B*2 allele	MBF1 [T00492]	141	151	14.636473	CTCAAAAAAAA	0.06025	0.01851
LAPTM4B*2 allele	MBF1 [T00492]	192	202	14.636473	GACTAAAAGAA	0.06025	0.01851
LAPTM4B*2 allele	STAT5A [T04683]	170	173	0	GGAA	2.03125	2.09129
LAPTM4B*2 allele	STAT5A [T04683]	185	188	0	GGAA	2.03125	2.09129
LAPTM4B*2 allele	STAT5A [T04683]	247	250	0	GGAA	2.03125	2.09129
LAPTM4B*2 allele	POU3F2 [T00630]	176	182	9.725533	AAATCGC	0.06348	0.04187
LAPTM4B*2 allele	POU3F2 [T00630]	305	311	0.252682	TGGATTT	0.19043	0.05446
LAPTM4B*2 allele	FOXP3 [T04280]	175	183	14.668224	AAAATCGCA	0.00793	0.00413
LAPTM4B*2 allele	c-Ets-2 [T00113]	184	191	2.827022	AGGAACTA	0.09521	0.07905
LAPTM4B*2 allele	c-Ets-2 [T00113]	246	253	4.629332	AGGAAGGC	0.14282	0.14249
LAPTM4B*2 allele	c-Myb [T00137]	186	193	13.847077	GAACTAGA	0.06348	0.03251
LAPTM4B*2 allele	c-Myb [T00137]	217	224	12.979731	TGGAGTTA	0.14282	0.07497
LAPTM4B*2 allele	AP-3 (2) [T00039]	194	200	11.290546	CTAAAAG	0.38086	0.20639
LAPTM4B*2 allele	HNF-3beta [T02513]	195	201	3.504129	TAAAAGA	0.31738	0.134
LAPTM4B*2 allele	HNF-3beta [T02513]	306	312	3.66377	GGATTTA	0.31738	0.134
LAPTM4B*2 allele	YY1 [T00915]	101	107	7.55849	CCATTGC	0.34912	0.35388
LAPTM4B*2 allele	YY1 [T00915]	213	219	8.35288	TGAATGG	0.31738	0.2638
LAPTM4B*2 allele	YY1 [T00915]	301	307	7.441476	CTGATGG	0.34912	0.35388
LAPTM4B*2 allele	YY1 [T00915]	395	401	3.973697	CACATGG	0.12695	0.10167
LAPTM4B*2 allele	Hlf [T01071]	221	229	6.667463	GTTACACGA	0.02579	0.01154
LAPTM4B*2 allele	Myf-3 [T00519]	234	241	2.45354	CCAGATGA	0.11108	0.07762
LAPTM4B*2 allele	Myf-3 [T00519]	336	343	0	GCAGCTGC	0.14282	0.20809
LAPTM4B*2 allele	Myf-3 [T00519]	355	362	0	GCAGCTGG	0.14282	0.20809
LAPTM4B*2 allele	MyoD [T00525]	234	241	2.45354	CCAGATGA	0.11108	0.07762
LAPTM4B*2 allele	MyoD [T00525]	336	343	0	GCAGCTGC	0.14282	0.20809
LAPTM4B*2 allele	MyoD [T00525]	355	362	0	GCAGCTGG	0.14282	0.20809
LAPTM4B*2 allele	NF-X3 [T01514]	235	242	5.226274	CAGATGAA	0.08728	0.07246
LAPTM4B*2 allele	NF-X3 [T01514]	475	482	8.793097	CAGAAGCT	0.14282	0.13528
LAPTM4B*2 allele	LCR-F1 [T01599]	238	243	5.346725	ATGAAA	0.63477	0.33882
LAPTM4B*2 allele	LCR-F1 [T01599]	375	380	0.029165	ATGACG	0.50781	0.33515
LAPTM4B*2 allele	p53 [T00671]	45	51	0.525867	GGGCAGG	0.06348	0.15461
LAPTM4B*2 allele	p53 [T00671]	118	124	8.914655	GGGCGAC	0.31738	0.69283
LAPTM4B*2 allele	p53 [T00671]	249	255	9.164941	AAGGCCC	0.31738	0.69283
LAPTM4B*2 allele	p53 [T00671]	269	275	7.959461	GGGCCGA	0.19043	0.36751
LAPTM4B*2 allele	p53 [T00671]	365	371	9.966389	GGAGCCC	0.12695	0.38348
LAPTM4B*2 allele	p53 [T00671]	407	413	7.700559	TCCGCCC	0.12695	0.24071
LAPTM4B*2 allele	p53 [T00671]	412	418	3.891597	CCCGCCC	0.19043	0.52463
LAPTM4B*2 allele	p53 [T00671]	508	514	3.624632	CAGGCCC	0.09521	0.20746
LAPTM4B*2 allele	EIIaE-A [T00246]	43	49	14.472869	TTGGGCA	0.8252	1.50151
LAPTM4B*2 allele	EIIaE-A [T00246]	66	72	10.118218	CCGGGAG	0.57129	1.10588
LAPTM4B*2 allele	EIIaE-A [T00246]	116	122	10.118218	CTGGGCG	0.57129	1.10588
LAPTM4B*2 allele	EIIaE-A [T00246]	167	173	8.709302	AAGGGAA	0.57129	1.22589
LAPTM4B*2 allele	EIIaE-A [T00246]	251	257	14.472869	GGCCCGG	0.8252	1.50151
LAPTM4B*2 allele	EIIaE-A [T00246]	267	273	10.118218	CAGGGCC	0.57129	1.10588
LAPTM4B*2 allele	EIIaE-A [T00246]	277	283	14.472869	TAGGGGA	0.8252	1.50151
LAPTM4B*2 allele	EIIaE-A [T00246]	290	296	8.709302	GAGGGTG	0.57129	1.22589
LAPTM4B*2 allele	EIIaE-A [T00246]	317	323	14.472869	CCGGGTG	0.8252	1.50151
LAPTM4B*2 allele	EIIaE-A [T00246]	367	373	13.063953	AGCCCGC	0.8252	1.64315
LAPTM4B*2 allele	EIIaE-A [T00246]	409	415	10.118218	CGCCCCG	0.57129	1.10588
LAPTM4B*2 allele	EIIaE-A [T00246]	414	420	8.709302	CGCCCCC	0.57129	1.22589
LAPTM4B*2 allele	EIIaE-A [T00246]	416	422	8.709302	CCCCCTC	0.57129	1.22589
LAPTM4B*2 allele	EIIaE-A [T00246]	420	426	14.472869	CTCCCCG	0.8252	1.50151
LAPTM4B*2 allele	EIIaE-A [T00246]	459	465	13.063953	AAGGGTA	0.8252	1.64315
LAPTM4B*2 allele	EIIaE-A [T00246]	510	516	13.063953	GGCCCGC	0.8252	1.64315
LAPTM4B*2 allele	NHP-1 [T00621]	256	262	9.83	GGACCTC	0.47607	0.38993
LAPTM4B*2 allele	NHP-1 [T00621]	376	382	9.83	TGACGTC	0.47607	0.38993
LAPTM4B*2 allele	NHP-1 [T00621]	377	383	9.83	GACGTCA	0.47607	0.38993
LAPTM4B*2 allele	NHP-1 [T00621]	390	396	6.571346	CGGGTCA	0.09521	0.12898
LAPTM4B*2 allele	TFIIB [T00818]	263	270	11.657154	CACTCAGG	0.2063	0.2925
LAPTM4B*2 allele	EBF [T05427]	263	273	6.8994	CACTCAGGGCC	0.00793	0.01932
LAPTM4B*2 allele	MZF-1 [T00529]	277	283	2.220412	TAGGGGA	0.12695	0.17016
LAPTM4B*2 allele	MZF-1 [T00529]	421	427	1.166538	TCCCCGT	0.12695	0.21649
LAPTM4B*2 allele	MZF-1 [T00529]	427	433	0.112664	TCCCCGC	0.12695	0.31845
LAPTM4B*2 allele	NF-kappaB1 [T00593]	279	290	14.056581	GGGGACTGGCGG	0.01271	0.02532
LAPTM4B*2 allele	Pax-5 [T00070]	295	301	7.762216	TGCACGC	0.06348	0.12759

LAPTM4B*2 allele	AhR:Arnt [T05394]	297	305	9.325207	CACGCTGAT	0.05356	0.05649
LAPTM4B*2 allele	OU2F2 (Oct-2.1) [T006	172	179	8.36845	AAGAAAAT	0.06348	0.0172
LAPTM4B*2 allele	OU2F2 (Oct-2.1) [T006	308	315	11.697988	ATTTACTC	0.05554	0.01943
LAPTM4B*2 allele	RAR-gamma [T00720]	311	318	5.085116	TACTCACC	0.06348	0.06732
LAPTM4B*2 allele	Myf-5 [T00521]	329	341	11.871492	AGCTCCAGCAGCT	0.01676	0.0256
LAPTM4B*2 allele	Myf-5 [T00521]	332	344	14.24579	TCCAGCAGCTGCT	0.0312	0.05065
LAPTM4B*2 allele	Myf-5 [T00521]	348	360	11.871492	AGCTCCAGCAGCT	0.01676	0.0256
LAPTM4B*2 allele	Myf-5 [T00521]	351	363	7.122895	TCCAGCAGCTGGC	0.00277	0.00412
LAPTM4B*2 allele	Myf-5 [T00521]	434	446	14.24579	CGCTGCAGCGGTC	0.0312	0.05065
LAPTM4B*2 allele	AP-4 [T00036]	333	343	2.654666	CCAGCAGCTGC	0.00471	0.00621
LAPTM4B*2 allele	AP-4 [T00036]	352	362	1.268101	CCAGCAGCTGG	0.00384	0.00573
LAPTM4B*2 allele	Tal-1 [T00790]	332	342	14.153038	TCCAGCAGCTG	0.04017	0.04655
LAPTM4B*2 allele	Tal-1 [T00790]	337	347	9.947032	CAGCTGCTTGG	0.01339	0.01658
LAPTM4B*2 allele	Tal-1 [T00790]	351	361	14.153038	TCCAGCAGCTG	0.04017	0.04655
LAPTM4B*2 allele	Tal-1 [T00790]	356	366	8.412013	CAGCTGGCTGG	0.02008	0.02862
LAPTM4B*2 allele	DP-1 [T01548]	365	374	14.492168	GGAGCCCGCG	0.05356	0.17372
LAPTM4B*2 allele	DP-1 [T01548]	425	434	12.705831	CGTCCCCGCC	0.05356	0.15075
LAPTM4B*2 allele	E2F-1 [T01542]	72	78	5.331496	GGCGGAG	0.12695	0.43536
LAPTM4B*2 allele	E2F-1 [T01542]	286	292	5.331496	GGCGGAG	0.12695	0.43536
LAPTM4B*2 allele	E2F-1 [T01542]	368	374	5.944817	GCCCGCG	0.12695	0.4156
LAPTM4B*2 allele	E2F-1 [T01542]	406	412	7.879274	GTCCGCC	0.31738	0.77859
LAPTM4B*2 allele	E2F-1 [T01542]	411	417	3.397038	CCCCGCC	0.19043	0.45057
LAPTM4B*2 allele	E2F-1 [T01542]	428	434	3.397038	CCCCGCC	0.19043	0.45057
LAPTM4B*2 allele	E2F-1 [T01542]	431	437	7.060495	CGCCGCT	0.12695	0.27655
LAPTM4B*2 allele	E2F-1 [T01542]	440	446	10.575502	AGCGGTC	0.12695	0.16594
LAPTM4B*2 allele	E2F-1 [T01542]	511	517	5.944817	GCCCGCG	0.12695	0.4156
LAPTM4B*2 allele	USF2b [T02377]	372	379	12.629773	GCGATGAC	0.57129	0.45763
LAPTM4B*2 allele	USF2b [T02377]	380	387	11.374018	GTCACGGA	0.28564	0.36021
LAPTM4B*2 allele	USF2b [T02377]	393	400	1.255756	GTCACATG	0.06348	0.04771
LAPTM4B*2 allele	c-Jun [T00132]	376	382	4.469954	TGACGTC	0.22217	0.19361
LAPTM4B*2 allele	c-Jun [T00132]	377	383	4.469954	GACGTCA	0.22217	0.19361
LAPTM4B*2 allele	c-Jun [T00132]	390	396	4.522753	CGGGTCA	0.22217	0.19361
LAPTM4B*2 allele	CREMalpha [T01803]	372	383	7.13467	GCGATGACGTCA	0.00781	0.00451
LAPTM4B*2 allele	CREMalpha [T01803]	376	387	5.668944	TGACGTCACGGA	0.0026	0.00192
LAPTM4B*2 allele	CREB [T00163]	375	383	0.032925	ATGACGTCA	0.00793	0.00573
LAPTM4B*2 allele	CREB [T00163]	376	384	0.024694	TGACGTCAC	0.00793	0.00573
LAPTM4B*2 allele	ATF-2 [T00167]	375	383	0.034254	ATGACGTCA	0.00793	0.00573
LAPTM4B*2 allele	ATF-2 [T00167]	376	384	0	TGACGTCAC	0.00793	0.00573
LAPTM4B*2 allele	ATF [T00051]	375	383	0.462475	ATGACGTCA	0.00992	0.0069
LAPTM4B*2 allele	ATF [T00051]	376	384	0.313871	TGACGTCAC	0.00992	0.0069
LAPTM4B*2 allele	E4F [T00222]	376	384	12.100443	TGACGTCAC	0.04761	0.02929
LAPTM4B*2 allele	c-Fos [T00123]	375	382	10.285697	ATGACGTC	0.12695	0.08854
LAPTM4B*2 allele	c-Fos [T00123]	377	384	11.816087	GACGTCAC	0.07141	0.04964
LAPTM4B*2 allele	c-Fos [T00123]	390	397	11.455199	CGGGTCAC	0.11108	0.11085
LAPTM4B*2 allele	ATF-1 [T00968]	374	384	2.828372	GATGACGTCAC	0.00174	0.00198
LAPTM4B*2 allele	ATF-1 [T00968]	375	385	3.669682	ATGACGTCACG	0.00446	0.00424
LAPTM4B*2 allele	Pax-2 [T01823]	373	379	3.495993	CGATGAC	0.15869	0.13025
LAPTM4B*2 allele	Pax-2 [T01823]	380	386	0.272056	GTCACGG	0.09521	0.11036
LAPTM4B*2 allele	Pax-2 [T01823]	393	399	5.131658	GTCACAT	0.31738	0.20576
LAPTM4B*2 allele	T3R-alpha1 [T01152]	386	396	13.79108	GACTCGGGTCA	0.08629	0.06956
LAPTM4B*2 allele	ER-beta [T04651]	388	396	9.122536	CTCGGGTCA	0.00793	0.00932
LAPTM4B*2 allele	RAR-beta [T00721]	389	396	2.799654	TCGGGTCA	0.05554	0.08434
LAPTM4B*2 allele	ER-alpha [T00261]	390	397	1.367305	CGGGTCAC	0.10315	0.10323
LAPTM4B*2 allele	NFI/CTF [T00094]	35	42	2.683003	TCGGTTGG	0.01587	0.02556
LAPTM4B*2 allele	NFI/CTF [T00094]	39	46	8.241664	TTGGTTGG	0.04761	0.05623
LAPTM4B*2 allele	NFI/CTF [T00094]	321	328	5.558661	GTGCTTGG	0.14282	0.12536
LAPTM4B*2 allele	NFI/CTF [T00094]	340	347	6.786076	CTGCTTGG	0.19043	0.18766
LAPTM4B*2 allele	NFI/CTF [T00094]	402	409	13.146004	CCAAGTCC	0.28564	0.16129
LAPTM4B*2 allele	Sp3 [T02338]	406	414	5.19869	GTCCGCCCC	0.0238	0.08976
LAPTM4B*2 allele	Sp3 [T02338]	411	419	0	CCCCGCCCC	0.00198	0.01404
LAPTM4B*2 allele	GCF [T00320]	409	419	5.946992	CGCCCCGCCCC	0.00335	0.02016
LAPTM4B*2 allele	MAZ [T00490]	412	422	14.391959	CCCGCCCCCTC	0.03645	0.15404
LAPTM4B*2 allele	MAZ [T00490]	418	428	1.454467	CCCTCCCCGTC	0.00434	0.01712
LAPTM4B*2 allele	MAZ [T00490]	424	434	14.561456	CCGTCCCCGCC	0.03645	0.15404
LAPTM4B*2 allele	WT1 [T00899]	414	424	7.524526	CGCCCCCTCCC	0.00397	0.02354
LAPTM4B*2 allele	WT1 [T00899]	425	435	11.883285	CGTCCCCGCCG	0.02678	0.10948
LAPTM4B*2 allele	R1 [T00711]	287	298	11.545139	GCGGAGGGTGCA	0.01339	0.03527
LAPTM4B*2 allele	R1 [T00711]	414	425	8.256952	CGCCCCCTCCCC	0.00967	0.02533
LAPTM4B*2 allele	LF-A1 [T00467]	7	13	9.701266	GGTGGCA	0.57129	0.77751
LAPTM4B*2 allele	LF-A1 [T00467]	43	49	1.561557	TTGGGCA	0.28564	0.50919

LAPTM4B*2 allele	LF-A1 [T00467]	470	476	8.920488	CCCGGCA	0.38086	0.53601
LAPTM4B*2 allele	LF-A1 [T00467]	503	509	5.631412	GGAGGCA	0.28564	0.33644
LAPTM4B*2 allele	GATA-1 [T00306]	495	500	1.908093	GGTATC	0.76172	0.37136
LAPTM4B*2 allele	WT1 I -KTS [T00900]	366	375	14.457704	GAGCCCGCGA	0.04662	0.19835
LAPTM4B*2 allele	WT1 I -KTS [T00900]	413	422	10.053856	CCGCCCCCTC	0.00793	0.04425
LAPTM4B*2 allele	WT1 I -KTS [T00900]	424	433	14.586256	CCGTCCCCGC	0.04662	0.19835
LAPTM4B*2 allele	WT1 -KTS [T01839]	366	375	14.457704	GAGCCCGCGA	0.04662	0.19835
LAPTM4B*2 allele	WT1 -KTS [T01839]	413	422	10.053856	CCGCCCCCTC	0.00793	0.04425
LAPTM4B*2 allele	WT1 -KTS [T01839]	424	433	14.586256	CCGTCCCCGC	0.04662	0.19835
LAPTM4B*2 allele	IA-1 [T05887]	413	425	6.673112	CCGCCCCCTCCCC	0.00101	0.00392
LAPTM4B*2 allele	ETF [T00270]	406	414	1.261043	GTCCGCCCC	0.02975	0.09859
LAPTM4B*2 allele	ETF [T00270]	411	419	0	CCCCGCCCC	0.00198	0.01404
LAPTM4B*2 allele	NF-1 [T00539]	401	405	0	GCCAA	0.50781	0.56301
LAPTM4B*2 allele	USF2 [T00878]	392	400	3.706299	GGTCACATG	0.0119	0.01097
LAPTM4B*2 allele	T3R-beta2 [T01350]	389	397	0.763705	TCGGGTCAC	0.00397	0.004
LAPTM4B*2 allele	ARP-1 [T00045]	388	396	3.301012	CTCGGGTCA	0.00793	0.0085
LAPTM4B*2 allele	E4F1 [T00223]	376	384	0.123447	TGACGTCAC	0.03174	0.03032
LAPTM4B*2 allele	E12 [T00204]	332	344	14.476348	TCCAGCAGCTGCT	0.00781	0.0123
LAPTM4B*2 allele	E12 [T00204]	351	363	14.548481	TCCAGCAGCTGGC	0.00781	0.0123
LAPTM4B*2 allele	TFII-I [T00824]	303	313	6.490932	GATGGATTTAC	0.00793	0.00749
LAPTM4B*2 allele	Pbx1b [T02087]	236	245	14.774452	AGATGAAAGA	0.05356	0.03264
LAPTM4B*2 allele	Pbx1b [T02087]	299	308	8.781358	CGCTGATGGA	0.02678	0.0106
LAPTM4B*2 allele	Arnt [T01346]	297	305	10.586083	CACGCTGAT	0.0238	0.03455
LAPTM4B*2 allele	AhR [T01795]	294	301	2.665859	GTGCACGC	0.11902	0.19485
LAPTM4B*2 allele	HMG I(Y) [T02368]	170	176	5.151271	GGAAGAA	0.15869	0.11562
LAPTM4B*2 allele	HMG I(Y) [T02368]	185	191	4.951498	GGAACTA	0.15869	0.11562
LAPTM4B*2 allele	HMG I(Y) [T02368]	247	253	7.014009	GGAAGGC	0.15869	0.28039
LAPTM4B*2 allele	STAT4 [T01577]	170	175	4.411765	GGAAGA	0.50781	0.64878
LAPTM4B*2 allele	STAT4 [T01577]	185	190	1.470588	GGAACT	0.50781	0.40202
LAPTM4B*2 allele	STAT4 [T01577]	247	252	5.882353	GGAAGG	0.12695	0.21649
LAPTM4B*2 allele	c-Ets-1 [T00112]	168	174	6.471844	AGGGAAG	0.12695	0.17596
LAPTM4B*2 allele	c-Ets-1 [T00112]	183	189	4.46609	AAGGAAC	0.15869	0.14819
LAPTM4B*2 allele	c-Ets-1 [T00112]	245	251	0.484609	AAGGAAG	0.09521	0.10632
LAPTM4B*2 allele	box binding factor [T00	239	248	8.695301	TGAAAGAAGG	0.05802	0.04399
LAPTM4B*2 allele	HSF1 (long) [T01042]	210	217	0	CCTTGAAT	0.0238	0.01528
LAPTM4B*2 allele	HSF1 (short) [T02104]	210	217	0	CCTTGAAT	0.0238	0.01528
LAPTM4B*2 allele	RFX1 [T01673]	79	87	7.500326	GTTGCAGTG	0.0238	0.03075
LAPTM4B*2 allele	HNF-1B [T01950]	22	29	14.384751	TAATCCCA	0.11108	0.05074
LAPTM4B*2 allele	NFdeltaE3A [T00976]	4	13	13.153012	TGTGGTGGCA	0.01785	0.01622
LAPTM4B*2 allele	AML1a [T02256]	0	9	7.146755	CGGGTGTGGT	0.01438	0.01537

TableS3. Primers for luciferase plasmids construction

Name		Sequence
LAPTM4B 5'UTR	Sense	5'-CTCGAGCCCTTGAATGGAGTTACACGAACG-3'
	Anti-sense	5'-AAGCTTCTGCCTCCTCGATACCCCGAGA-3'
LAPTM4B 5'UTR SOE-PCR	Sense	5'-CAGTCAACTGCTGGAGCCCGCGATGAC-3'
	Anti-sense	5'-CAGCAGTTGACTGGAGCTCCAAGCAC-3'

Table S4. The siRNA target sequences of AP4

Name	Sequence
TFAP4 scrambled nc	5’-GGCATCTTCGCAGTTACCT-3’
TFAP4 siRNA #5	5’-CACCATCGTGCAGGCAATCCA-3’
TFAP4 siRNA #4	5’-CAAGGAGAAGCTATTATATTT-3’
TFAP4 siRNA#2	5’-CCTGGGATTGTCAGCCTTCAA-3’

Table S5. The primer of AP4, LAPTM4B and GAPDH

	Sequence
AP4 forward	5’-GCAGGCAATCCAGCACAT-3’
AP4 reverse	5’-GGAGGCGGTGTCAGAGGT-3’
LAPTM4B forward	5’-AAGACCATTAGAAAGCACCAGG-3’
LAPTM4B reverse	5’-ACCAATCTAGGGCAGAACACTTA-3’
GAPDH forward	5’-ACGGATTTGGTCGTATTGGGCG-3’
GAPDH reverse	5’-CTCCTGGAAGATGGTGATGG-3’

Table S6. Antibodies used in WB

Name	Company and ID
anti-pAkt	CST4060
anti-pGSK3	CST5558
anti-Akt	CST9272
anti-c-Myc	CST5605
anti-Cyclin E1	CST4129
anti-p27	CST3686
anti-N-cadherin	CST13116
anti-p21	CST2947
anti-Caspase-3	CST9662
anti-PARP	CST9532
anti-β-actin	CST3700
anti-E-cadherin	abcam40772
anti-p-c-myc(Thr58)	abcam28822
anti-Vimentin	abcam92547
anti-TFAP4	Santa Curz sc-18593
anti-LAPTM4B	ABGENT AP20870a

Table S7. Relationship between AP4 expression and clinicopathological features of HCC

Variables		Patients	AP4 expression		P [a]
			Low	High	
All cases		117			
Gender					0.659
	Male	90	39	51	
	Female	27	10	17	
Age					0.704
	＜50	49	22	27	
	≥50	68	27	41	
Cirrhosis					0.091
	Yes	64	22	42	
	No	53	27	26	
Viral status					0.064
	Yes	83	30	53	
	No	34	19	15	
Tumor size					0.039
	＜5cm	51	27	24	
	≥5cm	66	22	44	
Vessel carcinoma embolus					0.003
	Yes	58	16	42	
	No	59	33	26	
Histopathological differentiation					0
	WD	24	19	5	
	MD	61	23	38	
	PD	32	7	25	
TNM stage					0.001
	I-II	43	22	16	
	III-IV	74	22	52	
Serum AFP level					0.703
	＜7ng/ml	47	21	26	
	≥7ng/ml	70	28	42	
Recurrence					0.27
	Yes	27	14	13	
	No	90	35	55	

a. Chi-square test

Table S8. Relationship between LAPTM4B-35 expression and clinicopathological features of HCC

Variables		Patients	LAPTM4B expression		P [a]
			Low	High	
All cases		117			
Gender					0.189
	Male	90	40	50	
	Female	27	8	19	
Age					1
	＜50	49	20	29	
	≥50	68	28	40	
Cirrhosis					0.008
	Yes	64	19	45	
	No	53	29	24	
Viral status					0.041
	Yes	83	29	54	
	No	34	19	15	
Tumor size					0.009
	＜5cm	51	28	23	
	≥5cm	66	20	46	
Vessel carcinoma embolus					0
	Yes	58	14	44	
	No	59	34	25	
Histopathological differentiation					0
	WD	24	18	6	
	MD	61	22	39	
	PD	32	8	24	
TNM stage					0
	I-II	43	30	13	
	III-IV	74	18	56	
Serum AFP level					1
	＜7ng/ml	47	19	28	
	≥7ng/ml	70	29	41	
Recurrence					0.824
	Yes	27	12	15	
	No	90	36	54	

a. Chi-square test
